# NLRP6 deficiency expands a novel CD103^+^ B cell population that confers immune tolerance in NOD mice

**DOI:** 10.3389/fimmu.2023.1147925

**Published:** 2023-02-23

**Authors:** James A. Pearson, Jian Peng, Juan Huang, Xiaoqing Yu, Ningwen Tai, Youjia Hu, Sha Sha, Richard A. Flavell, Hongyu Zhao, F. Susan Wong, Li Wen

**Affiliations:** ^1^ Section of Endocrinology, Internal Medicine, School of Medicine, Yale University, New Haven, CT, United States; ^2^ Diabetes Research Group, Division of Infection and Immunity, School of Medicine, Cardiff University, Cardiff, United Kingdom; ^3^ Department of Bioinformatics & Data Science, School of Public Health, Yale University, New Haven, CT, United States; ^4^ Department of Immunobiology, School of Medicine, Yale University, New Haven, CT, United States; ^5^ Howard Hughes Medical Institute, Chevy Chase, MD, United States

**Keywords:** inflammasome, type 1 diabetes (T1D), non-obese diabetic (NOD) mice, microbiome, NLRP6

## Abstract

**Introduction:**

Gut microbiota have been linked to modulating susceptibility to Type 1 diabetes; however, there are many ways in which the microbiota interact with host cells, including through microbial ligand binding to intracellular inflammasomes (large multi-subunit proteins) to initiate immune responses. NLRP6, a microbe-recognizing inflammasome protein, is highly expressed by intestinal epithelial cells and can alter susceptibility to cancer, obesity and Crohn’s disease; however, the role of NLRP6 in modulating susceptibility to autoimmune diabetes, was previously unknown.

**Methods:**

We generated NLRP6-deficient Non-obese diabetic (NOD) mice to study the effect of NLRP6-deficiency on the immune cells and susceptibility to Type 1 diabetes development.

**Results:**

NLRP6-deficient mice exhibited an expansion of CD103^+^ B cells and were protected from type 1 diabetes. Moreover, NLRP6-deficient CD103^+^ B cells express regulatory markers, secreted higher concentrations of IL-10 and TGFb1 cytokines and suppressed diabetogenic T cell proliferation, compared to NLRP6-sufficient CD103^+^ B cells. Microarray analysis of NLRP6-sufficient and -deficient CD103^+^ B cells identified 79 significantly different genes including genes regulated by lipopolysaccharide (LPS), tretinoin, IL-10 and TGFb, which was confirmed in vitro following LPS stimulation. Furthermore, microbiota from NLRP6-deficient mice induced CD103^+^ B cells in colonized NLRP6-sufficient germ-free mice; however, the long-term maintenance of the CD103^+^ B cells required the absence of NLRP6 in the hosts, or continued exposure to microbiota from NLRP6-deficient mice.

**Discussion:**

Together, our data indicate that NLRP6 deficiency promotes expansion and maintenance of a novel TGF -dependent CD103^+^ Breg population. Thus, targeting NLRP6 therapeutically may prove clinically useful.

## 1 Introduction

The NLR family pyrin domain containing 6 (NLRP6) protein forms an inflammasome complex in human (1) and mouse (2, 3) cells, and is important in cleaving IL-1β and IL-18. NLRP6 is expressed in epithelial cells (2–6) and at a lower level in immune cells (1, 4, 6). NLRP6 functions range from modulating immunity to bacteria (3, 6–8) and viruses (9), to regulating metabolic disease (10) and inducing protection from cancer (4, 11). Little is known of the role of NLRP6-expressing immune cell types in autoimmune diabetes. NLRP6-associated effects are attributed to modulation of the gut microbiota composition (2, 3, 10). However, other studies suggested that the altered microbiota composition may not be due to an intrinsic NLRP6-deficiency but extrinsic factors that include how the mice are reared for study (12, 13). Thus, understanding how NLRP6 modulates disease development is important.

B cells are antigen-specific antigen presenting cells (APCs), in addition to producing (auto)antibodies and importantly, regulating immune responses. Regulatory B cells (Bregs) are potent producers of IL-10, TGFβ and IL35 and have phenotypes found in marginal zone (precursor) B cells, CD1d^hi^CD5^+^ B cells, plasmablasts and plasma cells (14–18). In addition, microbial products such as lipopolysaccharide (LPS) (19–21), or microbiota-driven IL-1β and IL-6, produced by splenocytes (22), can induce Bregs, indicating the importance of microbial influences on Breg development.

In Type 1 diabetes (T1D), T cells destroy the beta cells; however, B cells are important in modulating disease development. In B cell-deficient non-obese diabetic (NOD) mice, a mouse model of human T1D, diabetes incidence is greatly reduced (23–25). Furthermore, B cell depletion immunotherapy in patients with T1D and NOD mice, helps preserve islet β-cells from T cell-mediated destruction and reduces autoimmunity (26, 27). B cells can also protect NOD mice from T cell-mediated autoimmunity (19, 21, 28). However, the role of inflammasomes in B cells in the immunopathogenesis of T1D has not been studied. In addition, to our knowledge, the role of NLRP6 in B cells, in any disease setting, has not been elucidated. Therefore, we investigated the role of B cells in NLRP6-deficient NOD mice and hypothesized that NLRP6 deficiency would alter B cell development and functions, and thus, modulate susceptibility to T1D.

## 2 Materials and methods

### 2.1 Mice

NOD/Caj mice have been maintained at Yale University for over 30 yrs. NLRP6-/- C57BL/6 mice (2) were backcrossed onto the NOD background for more than 10 generations, and the NOD genetic background was verified by Illumina mouse whole genome SNP scan (DartMouse^TM^, Hanover, NH, USA). All the mice studied were bred from homozygous breeders. Recombinase-activating-gene deficient NOD (Rag^-/-^NOD) mice, NY8.3 T cell receptor transgenic (TCR-Tg) NOD and BDC2.5 TCR-Tg NOD, obtained from the Jackson Laboratory, have been maintained at Yale University for nearly 20 years. TLR4-deficient NOD mice, (kindly provided by A.V. Chervonsky, University of Chicago) were maintained at Yale for over a decade. All mice were maintained in a 12-hr dark/light cycle, in specific pathogen–free (SPF) individually-ventilated filter cages with access to water and autoclaved food ad libitum at the Yale Animal Resource Center. Most mice were females at 12-16-weeks unless specified, and studied in the morning. Mice were randomly assigned to experiments from multiple breeders. Germ-free (GF) NOD mouse breeders were generously provided by A.V. Chervonsky (bred and maintained at the gnotobiotic facility of the Yale Animal Resource Center). The Yale University Institutional Animal Care and Use Committee approved the procedures used in this study.

### 2.2 Diabetes monitoring

Mice were monitored weekly for glycosuria with glucose strips (Bayer, Whippany, NJ, USA) from 10-weeks-old until termination (up to 30-weeks-old). Diabetes was confirmed following two consecutive positive glycosuria tests, 24-hrs apart, with a blood glucose ≥250mg/dl (13.9mmol/L), measured using a FreeStyle glucose meter (Abbott, Chicago, IL, USA).

### 2.3 Intestinal immune cell isolation

Peyer’s patches were first removed from the small intestine (duodenum, jejunum and ileum) and large intestine (cecum and colon). The intestinal sections were flushed with 1xPBS, opened longitudinally, then cut into 1cm sections, washed in PBS, followed by incubation in RPMI-1640 +1mM Dithiotriol (DTT) for 10mins (both Sigma-Aldrich (St Louis, MI, USA)). Cells were pelleted and suspended in HBSS (Calcium- and Magnesium-free) containing 25mM HEPES, 1mM DTT and 1mM EDTA (all Sigma-Aldrich), followed by incubation for 30 minutes at 37°C on a shaker at 140 rpm. Post-incubation, cells were vortexed vigorously for 10 seconds and filtered through a 100μm nylon membrane, to obtain the intestinal epithelial lymphocytes (IELs). Remaining tissues were further digested for 1hr at 37°C, with shaking at 250 rpm, in RPMI-1640 containing 1mg/ml Collagenase from Clostridium Histolyticum (Sigma-Aldrich). Post-incubation, cells were vortexed vigorously for 10 seconds and nylon membrane-filtered, to obtain the lamina propria lymphocytes (LPs). IELs and LPs were washed twice and resuspended in 30% Ficoll (GE HealthCare, Chicago, IL, USA), then layered on a 70% Ficoll solution prior to density centrifugation (469 xg, 20 mins, room temperature). Isolated IELs were washed and resuspended in RPMI complete media (RPMI-1640 + L-glutamine containing 5% FBS, 1mM HEPES, 1x MEM NEAA, 1mM Sodium pyruvate, 50μM 2-mercaptoethanol and 25μg Gentamycin sulfate (all Sigma-Aldrich)).

### 2.4 Islet histology analysis

Formalin-fixed pancreata were embedded in paraffin and stained with hematoxylin and eosin. Insulitis was scored under a light microscope. 110-140 islets from 5 mice were individually blind scored.

### 2.5 Pancreatic immune cell isolation

The pancreas was inflated with 3ml cold collagenase (Sigma; St Louis, MO, USA) solution (0.3mg/ml) through the bile duct with a 20G needle, then dissected and transferred into a 2 ml collagenase solution (1 mg/ml) in a siliconised glass tube followed by at 37°C in a water bath for 12–15 min. Following 3 washes, islets were hand-picked under a light microscope. Islets were treated with Cell Dissociation Solution (Sigma) and the single-cell suspension was harvested for flow cytometry.

### 2.6 Cell staining and flow cytometry

Cells were pre-incubated with a Fc-blocking mAb (2.4G2, Biolegend), then incubated with pre-titrated antibody combinations for 30mins at 4°C, washed and stored at 4°C until analysis. For intracellular staining, cells were stimulated with PMA (50ng/ml, Sigma-Aldrich) and Ionomycin (500ng/ml, Sigma-Aldrich) in the presence of 1μl/ml GolgiPlug™ (BD, Franklin Lakes, NJ, USA) for 4-hrs. Post-stimulation, cells were stained for surface makers as above, then washed before fixing (20mins at RT in the dark) and permeabilization (eBioscience™ intracellular fixation and permeabilization buffer kit). Cells were then incubated with a Fc-blocking antibody (2.4G2; 15 minutes, 4°C), prior to staining for intracellular markers (30mins, 4°C). Samples were analyzed on a BD LSRFortessa Flow Cytometer with Fluorescent minus one (FMO) controls and isotype controls for gating. Results were analyzed by FlowJo v10.4 (BD). Antibodies to B220 (RA3-6B2; AB_313007), CD1d (K253; AB_10643277), CD4 (GK1.5; AB_493647), CD8 (53-6.7; AB_312751), CD5 (53-7.3; AB_312735), CD11b (M1/70; AB_755986), CD11c (N418; AB_493568), CD19 (6D5; AB_439718), CD21/35 (7E9; AB_940405 or AB_940413), CD23 (B3B4; AB_312831), CD24 (M1/69; AB_439716), CD40 (FGK45; AB_2860731), CD44 (IM7; AB_312957), CD80 (16-10A1; AB_313127), CD86 (GL-1; AB_493342), CD103 (2E7; AB_2128621), CD138 (281-2; AB_10962911), IgA (mA-6E1; AB_465917; eBioscience, San Diego, CA, USA), TCRβ (H57-597; AB_893625), TLR4 (SA15-21; AB_2561874), I-A^K^ (cross reacts to I-A^g7^; 10-3.6; AB_313457), H2-K^d^ (SF1-1.1; AB_313741), IL-4 (11B11; AB_493320), IL-6 (MP5-20F3; AB_10694868), IL-10 (JES5-16E3; AB_2566331), IL-17A (TC11-18H10.1; AB_315464), IL-21 (MHALX21; eBioscience; AB_2784739), IFNγ (XMG1.2; AB_2295770), TNFα (MP6-XT22; AB_493328) and TGFβ (TW7-16B4; AB_10898159) were used, in addition to a Zombie Aqua™ fixable viability dye. All antibodies were purchased from BioLegend (San Diego, CA, USA) unless specified.

### 2.7 B cell isolation

Purified splenic B cells were isolated by magnetic (negative) selection using an EasySep™ Mouse B cell Isolation kit (StemCell Technologies, Vancouver, Canada), with >98% purity routinely confirmed by flow cytometry. CD103^+^ and CD103^-^ B cells were gated from live, single CD19^+^ cells after gating out TCRβ^+^, CD11b^+^ and CD11c^+^ cells, followed by gating on CD103^+^ and CD103^-^ cells and purification by Fluorescent-activated cell sorting (FACS).

### 2.8 T cell isolation

Total splenocytes were incubated with hybridoma supernatants containing mAbs to CD8 (TB105; for isolation of CD4^+^ T cells) or CD4 (GK1.5; for isolation of CD8^+^ T cells), together with MHC-II (10.2.16) for 30mins at 4°C (kindly provided by the late Charles Janeway (Yale University). After washing with PBS, the cells were further incubated for 45mins on ice with magnetic beads (QIAGEN (Hilden, Germany) conjugated with goat anti-mouse IgG and IgM (to remove B cells) or goat anti-rat IgG (to remove CD4^+^ or CD8^+^ T cells and MHC-II^+^ cells). CD4^+^ or CD8^+^ T cells were then separated using a magnetic plate with a purity >90-95% as verified by flow cytometry.

### 2.9 Adoptive transfer

Purified splenic T cells from diabetic (<1-week post-diagnosis) NOD mice were isolated and co-transferred (10^7^) with purified splenic total or CD103^-^ B cells (7.5x10^6^), from 12-16-week-old non-diabetic mice, intravenously into 4-week-old Rag-/-NOD mice.

### 2.10 Proliferation

Purified B cells (10^5^) were stimulated with either anti-mouse IgM (VWR International, Radnor, PA, USA) in the presence of anti-CD40 (FGK4.5, BioXcell, Lebanon, NH, USA)) or LPS from *E.coli* O111:B4 (Sigma-Aldrich). For T-B cell co-culture (10^5^/each), the splenic B cells were isolated and mitomycin-c-treated (Sigma-Aldrich), prior to co-culture with T cells in the presence of IGRP peptide for NY8.3 transgenic CD8^+^ T cells (VYLKTNVFL (29);) or BDC mimotope peptide for BDC2.5 transgenic CD4^+^ T cells (RTRPLWVRME (30);). After 48-hrs at 37°C, culture supernatants were collected prior to adding ^3^H-Thymidine, then a further 18-hr incubation was followed by harvest and analysis on a β-counter (Perkin Elmer, Waltham, MA, USA). Proliferation was measured as ΔCPM (counts per minute) with the background proliferation without antigen subtracted.

### 2.11 Transwell

Purified splenic CD103^+^ B cells (10^5^) were cultured in the insert of the transwell plate, while mitomycin-c-treated APCs (10^5^) were directly co-cultured with BDC2.5 transgenic CD4^+^ T cells (10^5^), in the presence of BDC mimotope peptide in the lower chamber of the transwell plate. Cells were cultured for 48-hrs at 37°C prior to supernatant collection and ^3^H-Thymidine addition, and assessed as described above.

### 2.12 Cytokine ELISA

IL-10, IL-17a, IL-21 and TGFβ1 were measured using the ELISA MAX™ kit (BioLegend), Ready-SET-Go! ELISA kits (eBioscience) and mouse TGFβ-1 DuoSet ELISA kit (R&D Systems, Minneapolis, MN, USA), respectively, according to the protocols provided by the manufacturers and the results were analyzed on a microplate spectrophotometer (Perkin Elmer).

### 2.13 Microarray

RNA was extracted from FACS-sorted splenic CD103+ or CD103- B cells, followed by cRNA synthesis and whole genome microarray analysis (Yale Center for Genomic Analysis). GeneChip^®^ WT Plus Reagent Kit was used for sample preparation and ss-cDNA generation (Thermo Fisher Scientific, Waltham, MA, USA). 150 ng of total RNA were used for input. Affymetrix GeneChip Mouse Gene 2.0 ST arrays were washed using the GeneChip^®^ Fludics Station 450 and scanned with the GeneChip Scanner 3000. All the reactions and hybridizations were carried out according to the manufacturer’s protocol. Data were normalized to control probes and adjusted p values were calculated. Microarray analysis was conducted using Ingenuity Pathway Analysis (IPA) software (QIAGEN). For heat map generation, the data for each gene was normalized to 1 (i.e. all 8 samples analyzed for each gene gave a sum of 1). RNA microarray data are available at GEO accession number GSE224472.

### 2.14 Quantitative PCR

Intestinal tissues were stored in Trizol (Sigma-Aldrich) at -80°C, prior to RNA extraction and clean up using the RNeady Mini Kit (QIAGEN). Purified B cells were directly processed for RNA extraction. Equimolar concentrations of RNA were subsequently used for cDNA synthesis using an iScript™ cDNA Synthesis Kit (Bio-Rad, Hercules, CA, USA). qPCR was performed using an iQ5 qPCR detection system (Bio-Rad) according to the manufacturer’s instructions. The relative mRNA abundance was determined using the 2^−ΔΔCt^ method by normalization with the housekeeping gene GAPDH. All primer sequences are listed in **Supplementary Table 1**.

### 2.15 Fecal bacterial DNA extraction and sequencing

Fecal samples from 12-week-old mice were collected and stored at −20°C. Bacterial DNA was extracted as previously described (31). Briefly, fecal samples were resuspended in 300 µl TE buffer (10 mM Tris and 1 mM EDTA, pH 8) containing 7.5 µl SDS (0.5%) and 3 µl proteinase K (20 mg/ml) and incubated for 1 hour at 37°C (all Sigma-Aldrich). One volume of phenol/chloroform/isoamyl alcohol (25:24:1), 200 µl of 20% SDS, and 0.3 g of zirconium/silica beads (0.1 mm; BioSpec, Bartlesville, OK, USA) were added, and samples were mixed with a Mini bead-beater (BioSpec) for 2 min. The samples were then mixed with 820 µl phenol/chloroform/isoamyl alcohol (25:24:1) and centrifuged, and the aqueous layer was collected into a new tube. The bacterial DNA was precipitated with 0.6 volume of isopropanol, washed with 70% ethanol, air dried, and resuspended in 100 µl of sterile water. The V4 region of the bacterial 16S ribosomal gene was amplified from each DNA sample with barcoded broadly conserved bacterial primers (forward (5′-GTGCCAGCMGCCGCGGTAA-3′) and reverse primer (5′-GGACTACHVGGGTWTCTAAT-3′)). The PCR products were purified with a gel extraction kit (QIAGEN) and quantified with a spectrophotometer (Nanodrop), and equimolar amounts of each sample were pooled and pyrosequenced on an Ion Torrent personal genome machine sequencing system (Thermo Fisher Scientific). The results were analyzed using the QIIME software package (version 1.8) and UPARSE pipeline (version 7.0). After removing the primer sequences, the sequences were demultiplexed, quality filtered using QIIME, and further quality and chimera filtered in UPARSE pipeline. Operational taxonomic units (OTUs) were picked with 97% identity in UPARSE pipeline. In QIIME, the Greengenes reference database was used for taxonomy assignment, which was performed at various levels using representative sequences of each OTU. β-Diversity was calculated to compare differences between microbial community profiles, and the data are shown as a principal coordinate analysis (PCoA).

### 2.16 Intestinal permeability assay

Mice were fasted overnight before gavage with FITC-dextran (600 mg/kg; ~4000 MW, Sigma-Aldrich), with food supply restored 2-hrs post-gavage. 2-hrs later, serum was separated from blood samples by centrifugation (2300 xg, 5mins, room temperature (RT)), and diluted 1:1 in PBS in a 96-well plate. Serum FITC-dextran concentrations were determined using a fluorescence spectrophotometer (Perkin Elmer), with serum samples from non-FITC-dextran-gavaged mice used as baseline. Standard curves were generated using known concentrations of FITC-dextran diluted in control serum. Concentrations were determined using linear regression of a standard curve.

### 2.17 Endotoxin measurement

Lipopolysaccharide (LPS) endotoxin content in the serum samples were detected using Pierce LAL Chromogenic Endotoxin Quantitation Kit (Thermo Fisher Scientific), using the manufacturer’s protocol.

### 2.18 Immunoglobulin ELISA

Immunoglobulins in the culture supernatants or luminal gut were measured by direct ELISA. Briefly, samples or standards were used to coat the 96-well plate overnight at 4°C. After washing and blocking (1-hr, RT) with 1% BSA in PBS on a shaking platform (400 rpm), the samples were then incubated with AP–conjugated goat anti-mouse IgA, IgG or IgM (Southern Biotech, Birmingham, AL, USA) for 2-hrs RT on a shaking platform). Samples were subsequently washed and PNPP substrate (Sigma-Aldrich) was added. The reaction was stopped upon addition of 1M NaOH. Samples were analyzed on a microplate spectrophotometer (Perkin Elmer) at 405 nm (OD). Antibody concentrations were determined by linear regression of standard curves.

### 2.19 Oral gavage

Fresh fecal pellets were resuspended in sterile PBS and homogenized using a Mini bead-beater (30secs; BioSpec). Fecal material was then centrifuged for 2-mins at low speed (52xg) to remove dietary residue. The supernatant was transferred to a new tube and spun. Following 2 further repeats, the combined supernatant was centrifuged at 469xg to remove mammalian cells. Bacteria in the supernatant were pelleted by centrifugation at high speed (1876xg, 5 min) and resuspended in PBS. Bacterial colony forming units (CFUs) were determined by optical density (OD), pre-determined using an *E. coli* strain, with a spectrophotometer (Bio-Rad). Four-week-old GF NOD mice were colonized with 200μl sterile PBS containing 2x10^8^ CFUs of stool bacteria by oral gavage. Colonized mice were terminated 2-4 weeks after gavage.

### 2.20 *In vitro* LPS stimulation

Splenic B cells (5x10^6^) were isolated and stimulated with or without LPS at different concentrations, for 20mins at 37°C. The cells were then pelleted and RNA was extracted immediately for cDNA synthesis and qPCR.

### 2.21 *In vitro* TGFβ1 stimulation and blocking assays

Splenic B cells (5x10^6^) were stimulated with 10ng/ml LPS for 12-hrs in the presence of either TGFβ1 or anti-TGFβ1 (BioXCell, clone 1D11.16.8; or a control antibody) at different concentrations. CD103 expression was evaluated by flow cytometry.

### 2.22 Statistics

Statistical significance was determined using either a Gehan-Breslow-Wilcoxon test, a two-tailed Student’s T-test, a Two-way ANOVA or ANOSIM. All statistical analysis was conducted in GraphPad Prism V9, except microarray analysis which was analyzed using Ingenuity Pathway Analysis.

## 3 Results

### 3.1 NLRP6-deficient B cells exhibit increased CD103 expression, and are associated with protection from both spontaneous and transferred diabetes

**Figure 1:**
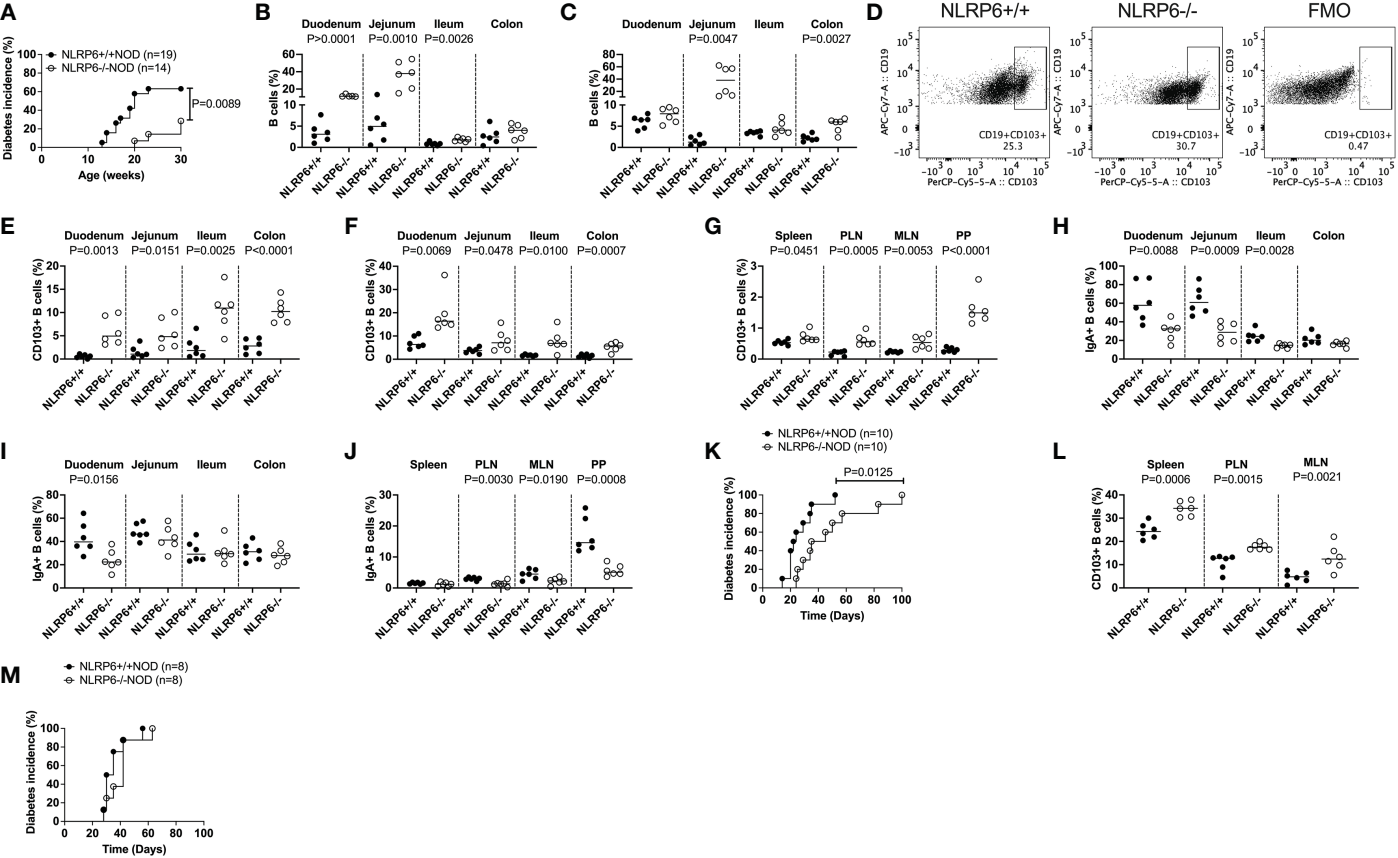
Increased CD103^+^ B cells, found in NLRP6-deficient mice exhibit are associated with protection from autoimmune diabetes development. **(A)** NLRP6+/+NOD (n=19) and NLRP6-/-NOD (n=14) female mice were observed for spontaneous diabetes development until 30-weeks-old. **(B, C)** The proportion of B cells were investigated by flow cytometry in the intestinal epithelial layer **(B)** or lamina propria **(C)** from 12-16-week-old NLRP6+/+NOD and NLRP6-/-NOD mice. B cells were gated from single, live CD45^+^TCRβ^-^CD11b^-^CD11c^-^ cells prior to gating on CD19^+^ cells. **(D)** Representative flow cytometric plots of CD103^+^ B cells, gated from single, live CD45^+^CD19^+^TCRβ^-^CD11b^-^CD11c^-^ cells prior to gating on CD103^+^ B cells. **(E-G)** CD103^+^ B cells from 12-16-week-old NLRP6+/+NOD and NLRP6-/-NOD mice from the intestinal epithelial layer **(E)**, lamina propria **(F)** and peripheral lymphoid tissues **(G)**. **(H-J)** Proportion of IgA^+^ B cells from the intestinal epithelial layer **(H)**, lamina propria **(I)** and peripheral lymphoid tissues **(J)**. **(K, L)** Splenic B cells from 12-16-week-old NLRP6+/+NOD and NLRP6-/-NOD mice were co-transferred with T cells from newly diabetic NLRP6+/+ NOD mice into 4-6-week old Rag-/-NOD mice (7.5x10^6^ B cells and 10x10^6^ T cells). **(K)** The incidence of diabetes development in the Rag-/- NOD recipients (n=10/group). **(L)** CD103^+^ B cell proportion and number in non-diabetic Rag-/- NOD mice, 2-weeks after transfer. **(M)** The incidence of diabetes development in the Rag-/- NOD recipients of splenic CD103^-^ B cells either from 12-16-week-old NLRP6+/+NOD or NLRP6-/-NOD mice co-transferred with T cells from newly diabetic NLRP6+/+ NOD mice (7.5x10^6^ CD103^-^ B cells and 10x10^6^ T cells). Abbreviations include pancreatic lymph nodes (PLN), mesenteric lymph nodes (MLN), Peyer’s patches (PP) and Fluorescence minus one (FMO). Data are pooled from 2-3 independent experiments (n=6 unless specified) with lines indicating the median value. Data were assessed for significance using a Gehan-Breslow-Wilcoxon test **(A, K, M)** or a two-tailed Student’s T-test.

To explore the role of NLRP6 in T1D, we generated NLRP6-/-NOD mice, backcrossing NLRP6-/-C57BL/6 mice onto the NOD genetic background for over 10 generations with 99.2% NOD purity. We observed NLRP6-/-NOD and NLRP6+/+ littermates for spontaneous diabetes, and observed significantly delayed and reduced T1D development in female NLRP6-/- mice with reduced immune infiltration of the pancreas (**Figure 1A** and **Supplementary Figure 1A**) compared to NLRP6+/+ mice. Additionally, we studied mice at 12-16-weeks old, when some NLRP6+/+NOD, but not NLRP6-/-NOD mice, had developed diabetes. Whilst NLRP6 in intestine is defined (2–5, 11), the role of NLRP6 in the immune cells within the intestinal tissues is not clear. We observed increased intestinal B cells in both the intestinal epithelial layer and lamina propria (LP) of the small and large intestine in NLRP6-/- mice, compared to NLRP6+/+ mice (**Figures 1B, C**); however, the B cell proportion in other peripheral lymphoid tissues was comparable between the NLRP6-/- and NLRP6+/+ mice (**Supplementary Figure 1B**). Further phenotypic analysis of the B cells revealed an expanded CD103^+^ B cell population in NLRP6-/- mice in all intestinal and peripheral lymphoid tissues studied, except the pancreas (**Figures 1D-G** and **Supplementary Figure 1C**). This may suggest a dominant role of these CD103^+^ B cells in limiting entry of immune cells to the pancreas. Similarly, we also observed an increase in CD103 median fluorescent intensity (MFI) in all tissues examined from NLRP6-/- mice, compared to NLRP6+/+ mice (**Supplementary Figures 1D-F**). Interestingly, we also found differences in the intestinal and peripheral IgA^+^ B cells in NLRP6-/- mice (**Figures 1H-J**). Coinciding with the expansion of CD103^+^ B cells, CD11b^+^CD11c^+^, CD11c^+^CD11b^-^ dendritic cells (DCs) cell subsets and CD11b^+^CD11c^-^ macrophages were reduced in some intestinal and peripheral lymphoid tissues in NLRP6-/-NOD mice compared to NLRP6+/+NOD mice (**Supplementary Figures 2A-I**); however, no differences were seen in CD103^+^ DCs between NLRP6-/- or NLRP6+/+ mice in any tissues studied (**Supplementary Figures 2J-L**). We observed no differences in T cell or Treg proportion (**Supplementary Figure 3**). To determine if NLRP6-/- B cells contributed to β-cell immune tolerance, we co-transferred splenic B cells isolated from either NLRP6+/+ or NLRP6-/- donor mice with splenic T cells from newly diagnosed diabetic NOD mice (NLRP6+/+) into immunodeficient Rag-/-NOD mice. We found that NLRP6-/- B cells significantly delayed diabetes development in the Rag-/-NOD recipients, compared to the recipients that were co-transferred with NLRP6+/+ B cells (**Figure 1K**). Moreover, this delay was associated with an increased frequency of CD103^+^ B cells in NLRP6-/- B cell recipients (**Figure 1L**). To verify the role of CD103^+^ B cells in the protection, we repeated the experiment with CD103^+^ depleted B cells from both NLRP6+/+ or NLRP6-/- mice and found no difference in diabetes development (**Figure 1M**), suggesting CD103^+^ B cells delayed development of T1D. Together, our data indicated that NLRP6-/- CD103^+^ B cells were potentially associated with immune tolerance to autoimmune diabetes.

### 3.2 CD103^+^ B regulatory subsets are expanded in NLRP6-deficient mice

**Figure 2:**
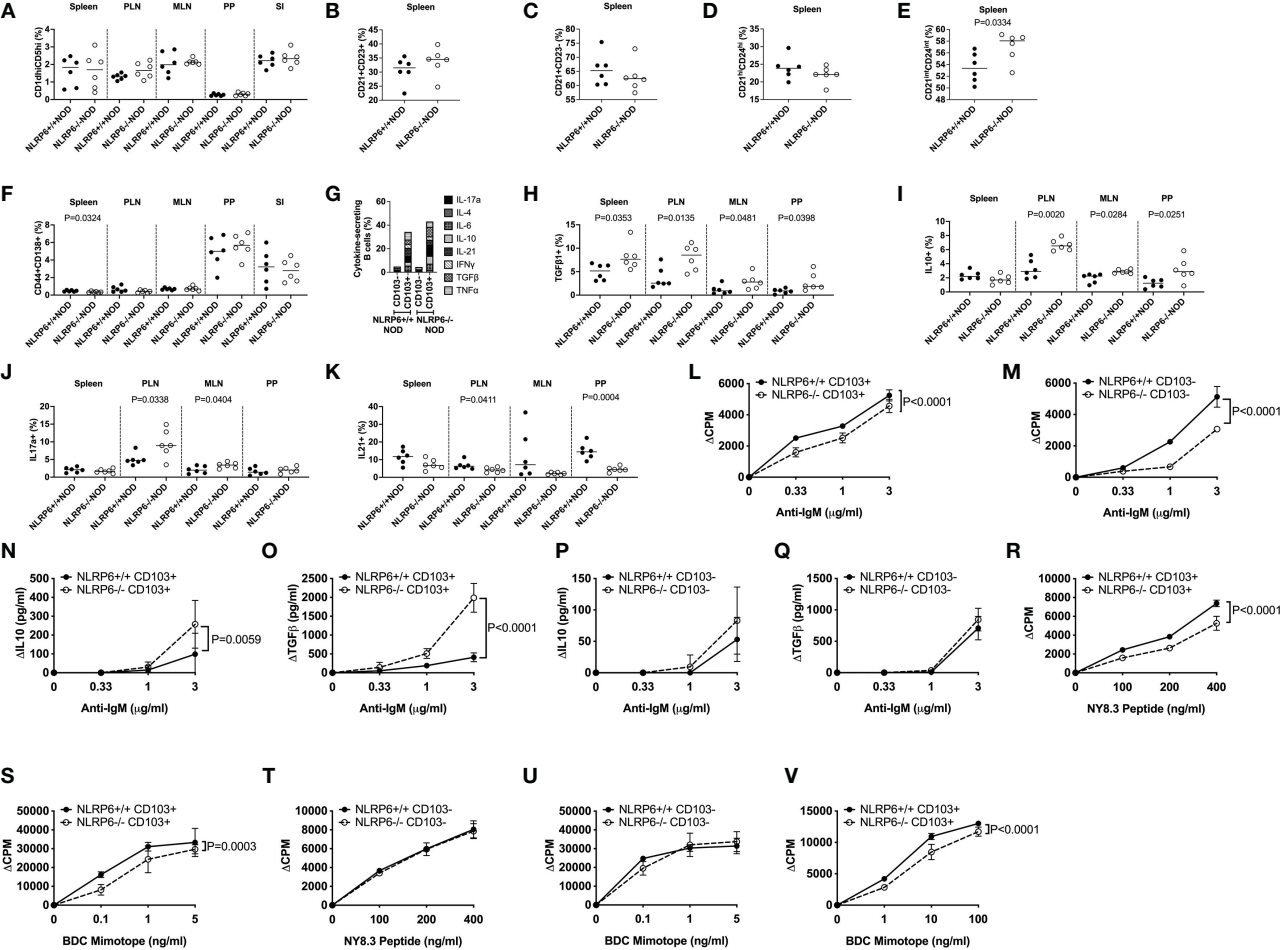
Characterization of CD103^+^ B cells and their function. CD103^+^ and CD103^-^ B cells from 12-16-week-old NLRP6+/+NOD and NLRP6-/-NOD mice were studied for phenotypic and functional changes. CD19^+^ B cells were gated from live, single CD19^+^TCRβ^-^CD11b^-^CD11c^-^ cells prior to gating on CD103^+^ cells and subsequent gating on the specific regulatory B cell subsets. Proportion of CD1d^hi^CD5^hi^ **(A)**, splenic CD21^+^CD23^+^ **(B)**, splenic CD21^+^CD23^-^ **(C)**, splenic CD21^hi^CD24^hi^ **(D)**, splenic CD21^int^CD24^int^ **(E)** and CD138^+^CD44^+^ **(F)** B cells. CD103^+^ B cell secreted cytokines were investigated following brief 4-hr PMA and Ionomycin stimulation in the presence of Golgi Plug. Cytokine-secreting B cells were gated from live, single CD19^+^TCRβ^-^CD11b^-^CD11c^-^CD103^+^ cells prior to gating on the specific cytokine. **(G)** Summarised cytokine results from the PLN of all cytokines investigated in both CD103^+^ and CD103^-^ B cells from NLRP6-sufficient and -deficient mice. Individual CD103^+^ B cell secreted cytokines are shown for TGFβ1- **(H)**, IL-10- **(I)**, IL-17a- **(J)** and IL-21 **(K)** from all tissues studied. CD103^+^ **(L)** or CD103^-^ **(M)** B cells were stimulated with anti-IgM (in the presence of 1μg/ml anti-CD40). Proliferation was assessed by ^3^H-Thymidine incorporation. Culture supernatants from **(L-M)** taken at 48-hrs were measured for IL-10 and TGFβ1 **(N-Q)** concentrations by ELISA. **(R-U)** CD103^+^ B cells **(R, S)** or CD103^-^ B cells **(T, U)** were mitomycin-c-treated and co-cultured 1:1 with NY8.3 CD8^+^ T cells or BDC2.5 CD4^+^ T cells in the presence of IGRP peptide (designated NY8.3 peptide) or BDC2.5 mimotope peptide (designated BDC mimotope) respectively, and assessed for proliferation by ^3^H-Thymidine incorporation, averaged from triplicates. **(V)** CD103^+^ B cells were cultured in a transwell with BDC2.5 CD4^+^ T cells and mitomycin-c-treated NOD splenocytes in the presence of BDC mimotope, and assessed for proliferation by ^3^H-Thymidine incorporation, averaged from triplicates. All data shown **(L-V)** were corrected for background (baseline proliferation CPM or cytokine production cytokine in the absence of stimulation). Abbreviations include pancreatic lymph nodes (PLN), mesenteric lymph nodes (MLN) and Peyer’s patches (PP). Data were pooled from 2-3 independent experiments (n=6-10), with lines indicating the median value **(A-K)** or mean and SD **(L-V)**. Data were assessed for significance using a Student’s T-test **(A-K)** or a Two-way ANOVA **(L-V)**.

We further examined the CD103^+^ B cells, comparing their phenotype with features commonly associated with Bregs. A number of Breg populations have been suggested, including CD1d^hi^CD5^hi^ B10 cells, splenic Transitional 2 (T2) marginal zone (MZ) precursor Bregs (CD21^+^CD23^+^), MZ Bregs (CD21^+^CD23^-^), antigen-experienced T2 MZ Bregs (CD21^int^CD24^int^) and plasmablast Bregs (CD44+CD138+) (14–17, 32). We found few proportional changes in any of these Breg subsets (**Figures 2A-F** and **Supplementary Figure 4**) between NLRP6+/+ and NLRP6-/- mice; however, comparison of the overall phenotype of CD103^+^ and CD103^-^ B cells between NLRP6-sufficient and NLRP6-deficient NOD mice demonstrated that NLRP6-deficient mice had more CD103^+^ than CD103^-^ Bregs than their wild-type counterparts (**Supplementary Figure 5**). In contrast, CD103^-^ B cells between NLRP6-sufficient and -deficient mice showed minimal differences (**Supplementary Figure 6**), suggesting that NLRP6-deficiency contributes to the increased CD103^+^ B cell population with several Breg phenotypes.

### 3.3 NLRP6-deficiency enhances CD103^+^ B cell cytokine secretion and reduces B cell and antigen-specific T cell proliferation

As CD103^+^ regulatory B cells were expanded, to assess the regulatory function of CD103^+^ B cells, we first determined the cytokine production profile by intracellular cytokine (ICC) staining. Interestingly, we found a significantly higher proportion of cytokine-secreting CD103^+^ B cells, compared with the CD103^-^ B cells (**Figure 2G** and **Supplementary Figure 7**). Furthermore, much higher proportions of NLRP6-/- CD103^+^ B cells were TGFβ1-, IL-10- and IL-17a-producing cells but IL-21-producing CD103^+^ B cells were reduced, compared to the CD103^+^ B cells from NLRP6+/+ mice (**Figures 2H-K**). No differences were found in IL-4-, IL-6-, IFNγ- or TNFα-producing CD103^+^ B cells with or without NLRP6 (**Supplementary Figures 8**, **9**). To further elucidate functional differences, we FACS-sorted splenic CD103^+^ and CD103^-^ B cells from NLRP6-sufficient and -deficient mice and stimulated the cells with anti-IgM (BCR) and anti-CD40 (coreceptor) *in vitro*. Both NLRP6-deficient CD103^+^ and CD103^-^ B cells had impaired proliferative responses to anti-IgM/CD40 stimulation, compared to their NLRP6-sufficient counterparts (**Figures 2L, M**). However, NLRP6-deficient CD103^+^ B cells secreted much higher concentrations of IL-10 and TGFβ1 (**Figures 2N-Q**). As B cells are also potent antigen presenting cells, we next assessed the CD103^+^ and CD103^-^ B cells for presentation of autoantigen to diabetogenic BDC2.5 CD4^+^ (recognizing insulin/chromogranin A hybrid peptide) and NY8.3 CD8^+^ (recognizing an islet-specific-glucose-6-phospatase catalytic subunit related protein) T cells. Interestingly, NLRP6-/- CD103^+^ B cells, but not NLRP6-/- CD103^-^ B cells, had significantly reduced ability to promote the antigen-specific proliferation of both diabetogenic CD4^+^ and CD8^+^ T cells, compared to the NLRP6-sufficient CD103^+^ and CD103^-^ B cells respectively (**Figures 2R-U**). There were no differences in MHC or the proportion of costimulatory molecule expression (**Supplementary Figure 10**); however, anti-inflammatory cytokine secretion by NLRP6-deficient CD103^+^ B cells, compared to NLRP6-sufficient CD103^+^ B cells, were sufficient to reduce antigen-specific T cell activation when cultured in a transwell system with BDC2.5 CD4^+^ T cells (**Figure 2V**). Together, our data suggested that the NLRP6-deficiency led to an expansion of novel CD103^+^ Breg cells that have enhanced tolerogenic cytokine production which dampens autoreactive T cell responses.

### 3.4 NLRP6-deficient CD103^+^ B cells are intrinsically different from NLRP6-sufficient CD103^+^ B cells

**Figure 3:**
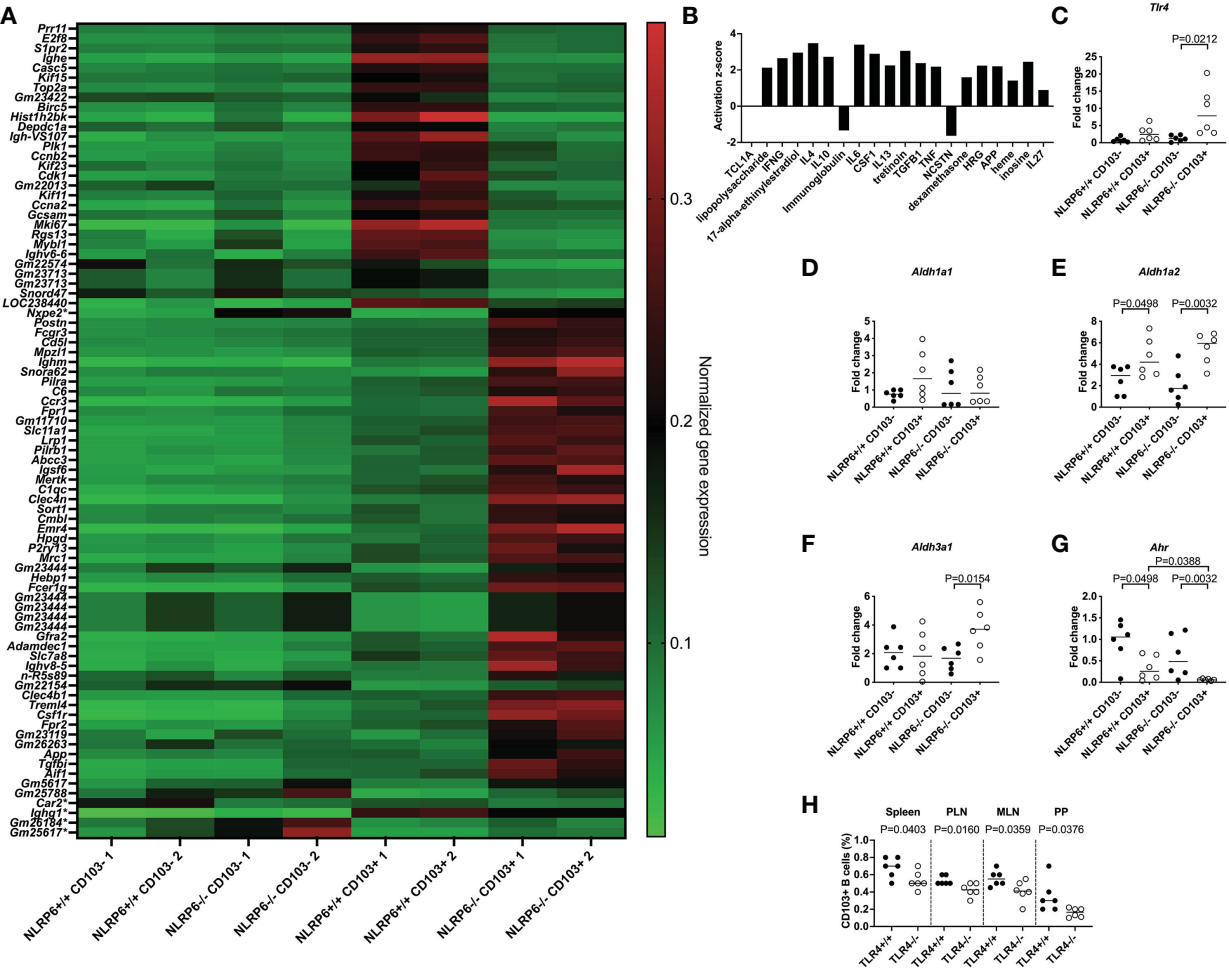
Identification of pathways involved in inducing regulatory NLRP6-deficient CD103^+^ B cells. FACS-sorted CD103^+^ and CD103^-^ B cells from 12-16-week-old NLRP6+/+NOD and NLRP6-/-NOD mice were investigated for gene expression changes by RNA microarray (n=2/group from 2 different experiments). **(A)** Heat map of normalized gene expression data showing the significant differences between CD103^+^ B cells from NLRP6+/+NOD and NLRP6-/-NOD mice (79 changes; top) or CD103^-^ B cells from NLRP6+/+NOD and NLRP6-/-NOD mice (5 changes marked with a *); 1 change shared with CD103^+^ B cell comparison (*Nxep2*)). High gene expression is shown in red, while green indicates low gene expression. Data are organized by genes increased or decreased in NLRP6+/+NOD vs NLRP6-/-NOD mice, in order of significance. **(B)** Upstream analysis using IPA software showing the activation z-score of the top 20 regulators of CD103^+^ B cells or CD103^-^ B cells using data from NLRP6-/-NOD CD103^+^ B cells vs CD103^-^ B cells. Data are organized from left to right in order of significance. **(C-G)** *Tlr4* **(C)**, *Aldh1a1* **(D)**, *Aldh1a2* **(E)**, *Aldh3a1* **(F)** and *Ahr* **(G)** gene expression from NLRP6-sufficient or -deficient CD103^-^ or CD103^+^ B cells, investigated by qPCR. The relative mRNA abundance was determined using the 2^−ΔΔCt^ method by normalization, with the housekeeping gene *Gapdh*. **(H)** Proportion of CD103^+^ B cells from TLR4-sufficient and TLR4-deficient NOD mice. Abbreviations include Toll-like receptor 4 (Tlr4), Aldehyde dehydrogenase (Aldh), Aryl hydrocarbon receptor (Ahr), pancreatic lymph nodes (PLN), mesenteric lymph nodes (MLN) and Peyer’s patches (PP). Data were pooled from 2-3 independent experiments (n=6), with the line indicating the median value. Data were assessed for significance using a two-tailed Student’s T-test **(C-H)**.

We investigated the molecular signature of the novel CD103^+^ Breg cells, especially the effect of NLRP6 on these cells, with an unbiased approach, performing an RNA microarray analysis of FACS-sorted CD103^+^ and CD103^-^ B cells from NLRP6+/+ and NLRP6-/- splenocytes. We observed 79 differences in gene expression between NLRP6-sufficient and -deficient CD103^+^ B cells, while only 5 genes were different between NLRP6-sufficient and -deficient CD103^-^ B cells (**Figure 3A**). In agreement with our earlier data at the cellular level, we also found that NLRP6-sufficient and -deficient CD103^+^ B cells were significantly different to the respective CD103^-^ B cells at the gene expression level (**Supplementary Figure 11A** and **Supplementary Table 2**). To determine potential regulators of CD103^+^ B cells we conducted an upstream pathway analysis using Ingenuity Pathway Analysis in NLRP6-deficient CD103^+^ B cells compared to NLRP6-deficient CD103^-^ B cells. Of the top 20 upstream regulators, we found multiple cytokines including IL-10 and TGFβ, all of which were secreted in higher concentrations from CD103^+^ B cells compared to CD103^-^ B cells (**Figure 3B** and **Figures 2H, I**). TGFβ, IL-6, 17-alpha-ethinylestradiol, LPS and IL-4 were also in the top 20 upstream regulators when comparing NLRP6-sufficient CD103^+^ to NLRP6-deficient CD103^+^ B cells (**Supplementary Figure 11B**). Interestingly, comparison between NLRP6-sufficient CD103^-^ and CD103^+^ B cells resulted in very different upstream regulators, suggesting different pathways of modulation were used (**Supplementary Figure 11C**). Our microarray data provided evidence that the absence of NLRP6 resulted in the promotion of tolerogenic CD103^+^ B cells in response to different upstream regulators, compared to CD103^-^ B cells and NLRP6-sufficient CD103^+^ B cells. In the comparisons between NLRP6-deficient CD103^+^ and CD103^-^ B cells (**Figure 3B**) and NLRP6-sufficient CD103^+^ and NLRP6-deficient CD103^+^ B cells (**Supplementary Figure 11B**), we identified LPS to be an upstream regulator, suggesting that gut microbiota may modulate CD103^+^ B cells.

### 3.5 TLR4 is upregulated in NLRP6-deficient CD103^+^ B cells

To test this hypothesis, we investigated *Tlr4* expression in different B cell subsets by qPCR, and our results revealed that *Tlr4* expression was significantly higher in NLRP6-deficient CD103^+^ B cells compared with CD103^-^ B cells (**Figure 3C**), suggesting an increased ability to detect bacterial LPS. This was also confirmed by flow cytometry (**Supplementary Figure 12**). Similar to CD103^+^ DCs in mice (33) and humans (34), we also found tretinoin (retinoic acid) was increased (**Figure 3B**). As retinal dehydrogenases convert retinal to retinoic acid, which in turn induces CD103 expression (35), we determined whether NLRP6 deficiency altered retinal dehydrogenase expression. We investigated 3 different retinal dehydrogenase isozymes that influence B cell development (36), in CD103^+^ and CD103^-^ B cells, by qPCR. Whilst we observed no significant changes in *aldh1a1* expression, *aldh1a2* expression was increased in CD103^+^ B cells (**Figures 3D, E**), in line with previous findings in CD103^+^ DCs (33). Whereas the enhanced *aldh1a2* expression in CD103^+^ B cells was independent of NLRP6 expression (**Figure 3E**), we found increased expression of *aldh3a1* in CD103^+^ B cells was NLRP6-dependent (**Figure 3F**). Aryl hydrocarbon receptor (AHR) activation induces *aldh3a1* (37) and promotes IL-10-producing Bregs (38). However, unexpectedly, we found that *Ahr* gene expression was significantly decreased in CD103^+^ B cells compared to CD103^-^ B cells, and the expression was even lower in NLRP6-deficient CD103^+^ B cells, compared to NLRP6-sufficient CD103^+^ B cells (**Figure 3G**). To confirm the potential importance of LPS for CD103^+^ B cell generation, we investigated CD103^+^ B cells from TLR4-deficient and TLR4-sufficient NOD mice. We found TLR4-deficient mice had reduced CD103^+^ B cells compared to TLR4-sufficient mice, confirming TLR4, the dominant receptor for LPS, was required for their expansion (**Figure 3H**). Together, these results of the altered gene expression of *Tlr4*, retinal dehydrogenases and *Ahr*, and TLR4-deficiency reducing the CD103^+^ B cell population, suggested that microbiota recognition and signaling may influence the development of the CD103^+^ Bregs in NLRP6-deficient mice.

### 3.6 Gut microbiota are influenced by NLRP6 deficiency

**Figure 4:**
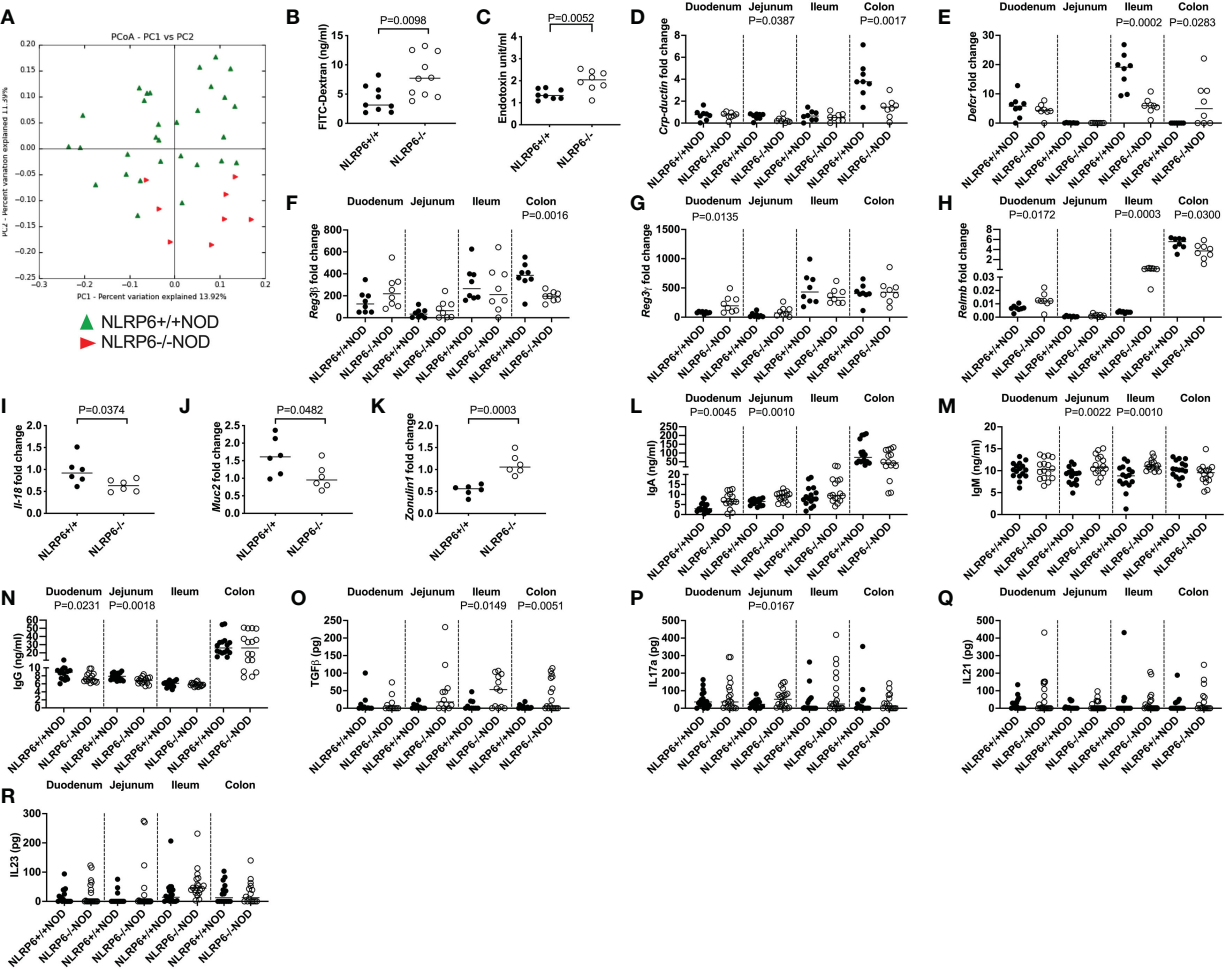
NLRP6-deficiency changes microbial β-diversity and promotes increased gut permeability, systemic microbial exposure and intestinal immunity. Microbial DNA was extracted from the fecal pellets of 12-week-old NLRP6^+/+^NOD (n=30; green) and NLRP6^-/-^NOD mice (n=8; red) and subjected to 16s rRNA deep sequencing **(A)**. β-diversity is shown as a principal component analysis. Intestinal permeability of 12-16-week old NLRP6+/+NOD (n=9) and NLRP6-/-NOD mice (n=10) following oral gavage of FITC-dextran **(B)**. Serum LPS Endotoxin measurements from NLRP6+/+NOD and NLRP6-/-NOD mice (n=8/group) measured by ELISA, with concentrations determined by linear regression **(C)**. *Crp-ductin* **(D)**, *Defcr* **(E)**, *Reg3β* **(F)**, *Reg3*γ **(G)**, *Relmb* **(H)**, *Il-18* **(I)**, *Muc2* **(J)** *and Zonulin-1* **(K)** gene expression from the intestinal tissue of 12-16-week old NLRP6-sufficient or -deficient mice conducted by qPCR (n=6-8). The relative mRNA abundance was determined using the 2^−ΔΔCt^ method by normalization with the housekeeping gene *Gapdh*. Intestinal IgA **(L)**, IgM **(M)**, IgG **(N)**, TGFβ **(O)**, IL-17a **(P)**, IL-21 **(Q)** and IL-23 **(R)** measured from the luminal flush of 12-16-week old mice by ELISA, with concentrations determined by linear regression (n=16-18). Data were assessed for significance using ANOSIM **(A)** or a two-tailed Student’s T-test **(B-R)**, with lines indicating the median value.

Previous reports suggested that altered microbiota in NLRP6-deficient C57BL/6 mice changed microbe-derived metabolites, which altered AHR activation and modulated NLRP6 inflammasome signaling in intestinal epithelial cells (2, 3, 10). Interestingly, the novel CD103^+^ Bregs identified in this study reside in highest proportion in the intestinal tissues (**Figures 1E, F**), compared to all other tissues studied (**Figure 1G**). Moreover, the highest expression of *Tlr4* was in NLRP6-/- CD103^+^ B cells (**Figure 3C**), suggesting a link between gut microbiota and CD103^+^ B cells. To investigate whether the microbiota could modulate CD103^+^ Breg development, we firstly studied the intestinal microbiota composition in the NLRP6-deficient and -sufficient mice. We found significant differences in the microbial β-diversity between NLRP6-sufficient and -deficient mice (12-weeks old, **Figure 4A**); however, no single microbial species was identified as significantly different, after performing multiple T-tests and corrections. However, we found that NLRP6-deficient mice had a more permeable intestine and a higher concentration of circulating LPS in the serum compared to NLRP6-sufficient mice (**Figures 4B, C**). Similar to previous studies, we found that the absence of NLRP6 altered the antimicrobial peptide expression in the small intestine, as well *as Il-18*, *Muc2* and *Zonulin1* gene expression (**Figures 4D-K**). We also identified that IgA and IgM antibodies were increased in the gut luminal fluid, whereas IgG antibodies were decreased in NLRP6-deficient mice compared to NLRP6-sufficient mice (**Figures 4L-N**). Furthermore, we detected increased concentrations of gut luminal TGFβ1 and IL-17a (**Figures 4O, P**), both of which were also increased in NLRP6-deficient CD103^+^ B cells (**Figures 2H, J**). There were no significant changes in intestinal IL-21 and IL-23 (**Figures 4Q, R**) or IL-10 (below detection level; data not shown). Together, our data suggested that the absence of NLRP6 altered intestinal immune responses, which may contribute to altering the microbial β-diversity, enhancing gut leakiness and increasing the circulating LPS.

### 3.7 Regulatory CD103^+^ B cells can be induced by gut microbiota from NLRP6-deficient mice but NLRP6-deficiency is required to maintain their regulatory function

**Figure 5:**
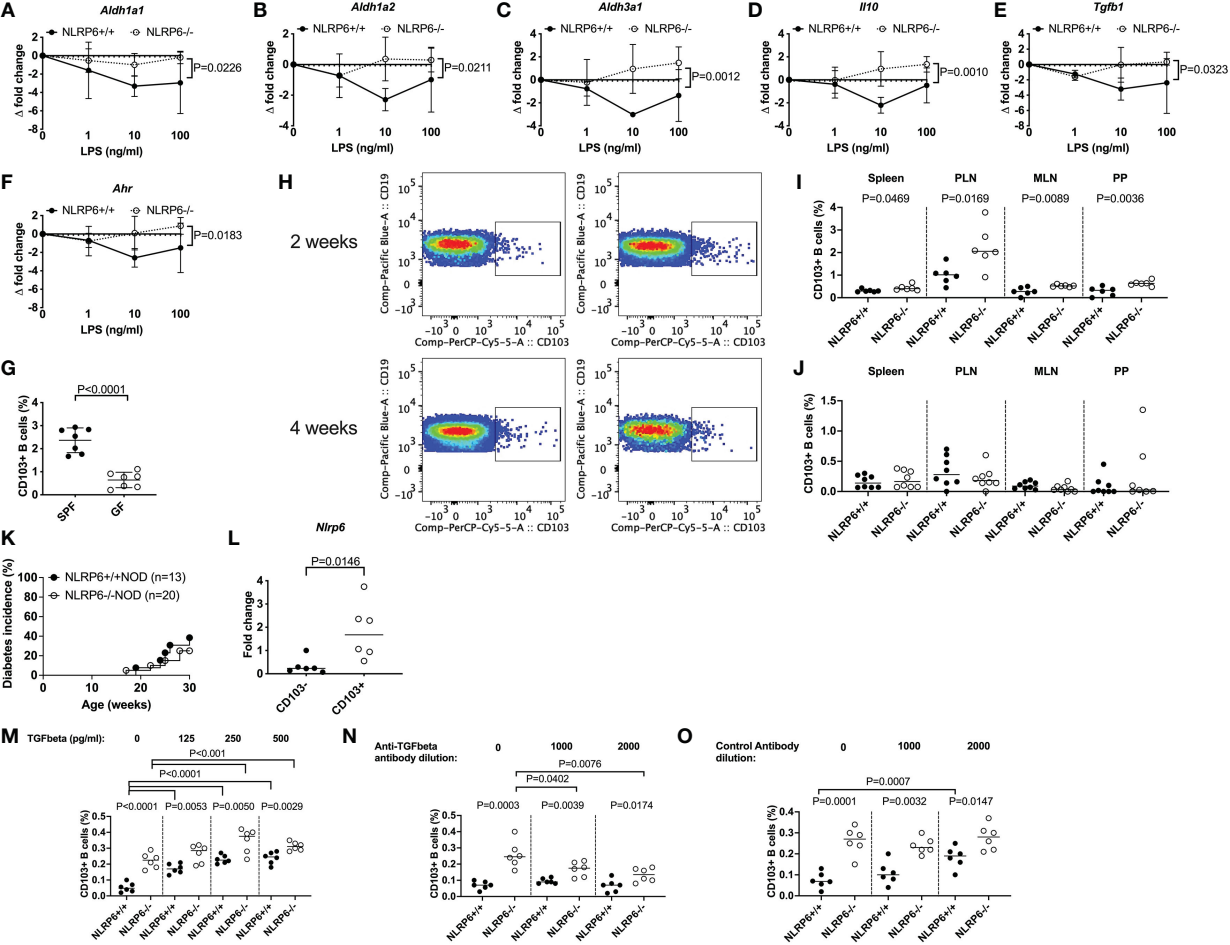
Gut microbiota, LPS and TGFβ1 induce regulatory NLRP6-deficient CD103^+^ B cells but a NLRP6 deficiency is required to maintain them. **(A-F)** *Aldh1a1* **(A)**, *Aldh1a2* **(B)**, *Aldh3a1* **(C)**, *Il-10* **(D)**, *Tgfβ1* **(E)** and *Ahr* **(F)** gene expression from NLRP6-sufficient or -deficient B cells following 20min LPS stimulation investigated by qPCR. The relative mRNA abundance was determined using the 2^−ΔΔCt^ method by normalization with the housekeeping gene *Gapdh*. **(G)** The proportion of CD103^+^ splenic B cells in SPF vs GF mice. **(H-J)** Isolated fecal microbiota was pooled from 12-16-week-old NLRP6+/+NOD and NLRP6-/-NOD mice (n=4) and transferred to germ-free NLRP6+/+NOD mice by oral gavage (2x10^8^ CFUs/recipient). Representative CD103^+^ B cell gating from bacterial-colonized mice **(H)** and summarized data from colonized mice 2- **(I)** and 4-weeks **(J)** post-gavage (NLRP6+/+ left column and NLRP6-/-NOD right column). **(K)** Diabetes incidence of cohoused NLRP6-sufficient and -deficient mice. **(L)** qPCR of the *Nlrp6* gene from CD103^+^ and CD103^-^ B cells from 12-16-week old NLRP6+/+NOD mice. **(M-O)** Proportion of CD103^+^ B cells post-overnight stimulation with LPS (10ng/ml) and TGFβ1 **(M)** or in the presence of an anti-TGFβ1 antibody **(N)** or control antibody **(O)**. Abbreviations include aldehyde dehydrogenase (aldh), aryl hydrocarbon receptor (ahr), specific pathogen-free (SPF), germ-free (GF), pancreatic lymph nodes (PLN), mesenteric lymph nodes (MLN) and Peyer’s patches (PP). Data were pooled from 2-3 independent experiments (n=6-8) with the exception of **K**, with lines representing the median value. Data were assessed for significance using a two-way ANOVA **(A-F)**, a two-tailed Student’s T-test **(I-N)** or a Gehan-Breslow-Wilcoxon test **(K)**.

We further investigated gut microbiota modulation of CD103^+^ B cells. As LPS was identified as an upstream regulator of NLRP6-deficient CD103^+^ B cells (**Figure 3B**), we stimulated total splenic B cells from NLRP6-deficient and NLRP6-sufficient mice with LPS *in vitro*, and assessed the gene expression by qPCR. We found that NLRP6-deficient B cells maintained or increased their regulatory profile in response to increasing LPS concentrations, as evidenced by a regulatory profile in *Aldh* isozyme, *Il-10*, *Tgfβ1* and *Ahr* expression, compared to both their own baseline (no stimulation) and NLRP6-sufficient B cells (**Figures 5A-F**). To confirm that microbiota promoted the development of CD103^+^ B cells, we compared splenic CD103^+^ B cells from specific pathogen-free (SPF; with microbiota) and germ-free (GF; without microbiota) NLRP6-sufficient mice. We found that GF mice had significantly fewer CD103^+^ B cells, suggesting that the presence of microbiota boosted the expansion of CD103^+^ B cells (**Figure 5G**).

To determine whether gut microbiota could induce CD103^+^ B cells *in vivo*, we performed fecal material transplant experiments by oral gavage of donor microbiota from SPF NLRP6-sufficient and -deficient mice into GF NLRP6-sufficient mice. We found that 2-weeks post-transfer, the recipients of NLRP6-deficient microbiota had increased proportions of CD103^+^ B cells compared to NLRP6-sufficient microbiota recipients (**Figure 5H (top), I**). However, NLRP6 deficiency was required to maintain the increased abundance of CD103^+^ B cells as the difference diminished by 4-weeks post-transfer (**Figure 5H (bottom), J**). To confirm whether continued NLRP6-deficient bacteria exposure influenced diabetes development, we co-housed NLRP6-sufficient and -deficient mice and observed their diabetes development. We found that NLRP6-sufficient NOD mice developed a similar low incidence of diabetes as NLRP6-deficient NOD mice (**Figure 5K**), suggesting that long-term exposure to NLRP6-deficient microbiota was able to protect NLRP6-sufficient mice from developing diabetes, compared to mice housed separately (**Figure 5K** vs. **Figure 1A**). To determine whether CD103^+^ B cells expressed NLRP6, we performed qPCR from FACS-sorted CD103^-^ and CD103^+^ B cells from non-cohoused NLRP6-sufficient mice. We found that CD103^+^ B cells had higher *Nlrp6* expression compared to CD103^-^ B cells (**Figure 5L**), suggesting that NLRP6 may have a direct effect on the CD103^+^ B cells. As TGFβ1 has been associated with CD103^+^ cell generation (33, 39), we investigated whether TGFβ1 induced CD103^+^ B cells. We found that TGFβ1 addition to LPS-stimulated B cells, induced CD103^+^ B cells in both NLRP6-sufficient and -deficient mice, with the latter maintaining a higher proportion (**Figure 5M**). In contrast, when an anti-TGFβ1 antibody was used, we found a reduction in NLRP6-deficient CD103+ B cells and no change in NLRP6-sufficient CD103+ B cells, which was not observed in the presence of a control antibody (**Figures 5N, O**). Together, our data suggest that the absence of NLRP6 may directly affect the development and maintenance of novel regulatory CD103^+^ B cells, induced through altered responses to microbiota and LPS with a requirement for TGFβ1 for their expansion. Importantly, these effects can be induced transiently in NLRP6-sufficient B cells when exposed to NLRP6-deficient dysbiotic microbiota, suggesting the potential for suppressing NLRP6 expression to boost Breg development.

## 4 Discussion

Our study has highlighted that, in the absence of NLRP6, CD103^+^ B cells, which have regulatory properties, are expanded in many lymphoid tissues and this novel subset of Breg contributes to immune tolerance to islet beta cell autoimmunity. CD103^+^ B cells were represented in cell subsets that express B10 Breg markers and secreted higher concentrations of IL-10 and TGFβ1. Moreover, LPS maintained and increased the regulatory function of the novel subset of CD103^+^ Bregs. Finally, we found that the gut microbiota from NLRP6-deficient mice induced peripheral CD103^+^ B cells in NLRP6-sufficient mice; however, the absence of NLRP6 was required to maintain the expansion of the CD103^+^ B cells, unless exposure to microbiota from NLRP6-deficient mice was continuous (e.g. cohousing of mice). Bregs are important immune modulators in autoimmune diseases, cancer and infections. Bregs are influenced by gut microbiota and gut microbiota-induced cytokines and AhR signaling (22, 38, 40), and here we have demonstrated that inflammasomes also modulate Breg development.

CD103 (integrin αE) is a surface glycoprotein and is highly expressed by a subset of DCs and regulatory T cells (Tregs). Our data showed that B cells also express CD103. However, the function of these CD103^+^ B cells was previously unknown. CD103 forms a complex with the β7 integrin (αEβ7). A deficiency in the β7 integrin significantly reduced B cell recruitment to the lamina propria (41, 42). We have found that CD103^+^ B cells were increased in the intestinal tissues, suggesting enhanced recruitment of CD103^+^ B cells to the intestine. In humans, CD103 expression on B cells is not typically seen (43); however, CD103^+^ B cells are expanded in hairy cell leukemia (HCL) and are used as a diagnostic marker, although their function was not clear (44). In our study, these cells promoted immune tolerance, and immune regulation by limiting pancreatic β-cell destruction by autoreactive T cells in type 1 diabetes. Our study suggests that NLRP6 deficiency leads to an expansion of CD103^+^ B cells, which can be induced by the gut microbiota from NLRP6-deficient mice. Thus, it is possible that the CD103^+^ B cell expansion observed in HCL patients may be modulated by gut microbiota and NLRP6 inflammasome signaling.

The role of gut microbiota in NLRP6-deficient mice is controversial, some studies have indicated their importance (2, 3, 10), while others did not find differences from NLRP6-sufficient mice (12, 13). We found altered β-diversity of the gut microbiota in NLRP6-deficient NOD mice but were unable to identify individual bacteria to which this change could be attributed, after statistical corrections. However, the absence of NLRP6 promoted a more permeable gut barrier, which may predispose the mice to type 1 diabetes development (45, 46) (as has also been suggested in humans (47, 48)); however, in contrast to published work, here, the mice with a more permeable gut were more protected from developing diabetes. We believe that the increased gut permeability in the NLRP6-deficient mice leads to increased LPS in the circulation, which protects NOD mice from diabetes development (19). Further, the increased gut permeability results in increased microbial exposure to the immune system and primes the Breg cells.

Interestingly, CD103^+^ B cells express NLRP6, albeit at a low level. Moreover, NLRP6 directly impacts on CD103^+ ^B cells, as shown by the altered response to LPS and upregulation of anti-inflammatory genes in the NLRP6-deficient B cells. The Ahr and associated genes have been clearly linked to anti-inflammatory responses and regulation in the immune system (49). The reduction of *Ahr* gene expression and the enhanced *Aldh3a1* gene expression in CD103^+^ B cells from NLRP6-deficient NOD mice suggest that Aldh3a1 may be regulated independently of Ahr; however, upon LPS stimulation, Aldh3a1 was upregulated which coincided with the recovery of the expression of Ahr (from baseline). Thus, our results suggest that LPS may activate both *Ahr* and *Aldh* gene expression upon stimulation. Furthermore, fecal microbiota from NLRP6-deficient mice were able to induce CD103^+^ B cell expansion in colonized NLRP6-sufficient germ-free mice. However, maintaining the expanded CD103^+^ B cells required the absence of NLRP6 in the host. Although no specific microbial species were identified, we have demonstrated the important functional changes of B cells in response to the microbiota. Whether this is due to the microbiota or microbial metabolites (3), remains to be further studied. Inosine, one of the upstream regulators of NLRP6-deficient CD103^+^ Bregs, can also be produced by the microbiota and is important in inhibition of severe autoimmunity (50), and improving the efficacy of checkpoint inhibitor therapy in colorectal cancer (51). Thus, this would be a logical future investigation.

A limitation of our study is that we have not directly demonstrated the reverse interaction, i.e., if CD103^+^ B cells modulate the gut microbiota and whether the interaction is dependent on NLRP6 expression from tissue cells, all of which will require to be tested in cell-specific NLRP6-deficient mice. We have provided evidence that NLRP6-deficient mice have altered intestinal antibody titers and this most likely contributes to the modulation of the gut microbiota, such as by antibody-coating of microbiota, known to have important implications in autoimmunity, including type 1 diabetes (52, 53). Future studies of cell-specific NLRP6-deficient mice will greatly aid in understanding the role of each cell type in regulation of the gut microbiota and whether NLRP6-deficient CD103^+^ B cells are reliant on cross-talk with other cells for their regulatory functions.

In summary, we present a novel regulatory B cell subset, characterized by the expression of CD103, increased in NLRP6 deficiency and are potent TGFβ- and IL-10 producers, all of which delayed and prevented diabetes development. Further investigation into the above upstream regulators and the small molecules associated with the microbial metabolites which enhance the regulatory function of CD103^+^ B cells may prove very valuable in devising future therapies to prevent autoimmune disease development.

## Data availability statement

The data presented in the study are deposited in the GEO repository, accession number GSE224472.

## Ethics statement

The animal study was reviewed and approved by Yale University Institutional Animal Care and Use Committee, Yale University.

## Author contributions

JAP, JP, JH, NT, YH and SS conducted the experiments. JAP, JP, XY and HZ analyzed the data. JAP, FW and LW designed the experiments. RF provided the mice for study. JAP, FW and LW wrote and edited the manuscript. All authors contributed to the article and approved the submitted version.

## Supplementary material

Supplementary Figure 1 NLRP6 deficiency alters B cell populations. Pancreas infiltration and B cells were investigated from intestinal and peripheral tissues of 12-16-week-old NLRP6+/+NOD and NLRP6-/-NOD mice by flow cytometry. **(A)** Histological summary of islet immune cell infiltration (n=5 mice; islet totals 110-140). **(B)** The proportion and number of B cells in peripheral tissues, gated from single, live CD45^+^TCRβ^-^CD11b^-^CD11c^-^ cells prior to gating on CD19^+^ cells. **(C)** The proportion of CD103^+^ B cells from the pancreas. **(D-F)** The median fluorescent intensity (MFI) of CD103 on B cells from the intestinal epithelial layer **(D)**, lamina propria **(E)** and peripheral tissues **(F)**. Abbreviations include pancreatic lymph nodes (PLN), mesenteric lymph nodes (MLN) and Peyer’s patches (PP). Data were pooled from 2 independent experiments (n=6), with lines indicating the median value. Data were assessed for significance using a Chi-square test **(A)** or a Student’s T-test.

Supplementary Figure 2 NLRP6 deficiency minimally alters other antigen-presenting cell populations. Antigen-presenting cells were investigated from intestinal and peripheral tissues of 12-16-week-old NLRP6+/+NOD and NLRP6-/-NOD mice by flow cytometry. **(A-C)** The proportion of CD11b^+^CD11c^+^ cells were investigated from the intestinal epithelial layer **(A)**, lamina propria **(B)** and peripheral tissues **(A)**. Cells were gated from single, live CD45^+^TCRβ^-^CD19^-^ prior to gating on CD11b^+^CD11c^+^ cells. **(D-F)** The proportion of CD11c^+^(CD11b^-^) cells were investigated from the intestinal epithelial layer **(D)**, lamina propria **(E)** and peripheral tissues **(F)**. Cells were gated from single, live CD45^+^TCRβ^-^CD19^-^ prior to gating on CD11b^+^CD11c^+^ cells. **(G-I)** The proportion of CD11b^+^(CD11c^-^) cells were investigated from the intestinal epithelial layer **(G)**, lamina propria **(H)** and peripheral tissues **(I)**. Cells were gated from single, live CD45^+^TCRβ^-^CD19^-^ prior to gating on CD11b^+^CD11c^-^ cells. **(J-L)** The proportion of CD103^+^CD11c^+^(CD11b^-^) cells were investigated from the intestinal epithelial layer **(J)**, lamina propria **(K)** and peripheral tissues **(L)**. Cells were gated as in **(D-F)** prior to gating on CD103^+^ cells. Abbreviations include pancreatic lymph nodes (PLN), mesenteric lymph nodes (MLN) and Peyer’s patches (PP). Data were pooled from 2 independent experiments (n=6), with lines indicating the median value. Data were assessed for significance using a Student’s T-test.

Supplementary Figure 3 NLRP6 deficiency does not alter T cell proportion or count. CD4^+^ and CD8^+^ T cells were investigated from the intestinal and peripheral tissues of 12-16-week-old NLRP6+/+NOD and NLRP6-/-NOD mice by flow cytometry. **(A-C)** The proportion of CD4^+^ T cells were investigated from the intestinal epithelial layer **(A)**, lamina propria **(B)** and peripheral tissues **(C)**. Intestinal cells were gated from single, live CD45^+^ cells prior to gating on TCRbeta^+^CD4^+^, while peripheral cells were gated from live CD45^+^ TCRbeta^+^CD19^-^ cells prior to gating on CD4. **(D-F)** The proportion of CD8^+^ T cells were investigated from the intestinal epithelial layer **(D)**, lamina propria **(E)** and peripheral tissues **(F)**. Cells were gated as above for CD4^+^ T cells, but on CD8^+^ T cells. **(G-I)** The proportion of CD4^+^CD25^+^FoxP3^+^ Tregs were investigated from the intestinal epithelial layer **(G)**, lamina propria **(H)** and peripheral tissues **(I)**. Cells were gated as above for CD4^+^ T cells prior to gating on CD25^+^FoxP3^+^ cells. Abbreviations include pancreatic lymph nodes (PLN), mesenteric lymph nodes (MLN) and Peyer’s patches (PP). Data were pooled from 2 independent experiments (n=6), with lines indicating the median value. Data were assessed for significance using a Student’s T-test.

Supplementary Figure 4 Gating of B cell subsets. CD19^+^ B cells were gated from live, single CD19^+^TCRβ^-^CD11b^-^CD11c^-^ cells prior to gating on either CD103+ or CD103- populations. Cells were then investigated for regulatory B10 cells (**A**; CD1d^hi^CD5^hi^), regulatory marginal zone B cells (**B, C**; CD21^+^CD23^+/-^, CD21^hi^CD24^hi^ and CD21^Int^CD24^int^) and plasmablast Breg populations (**D**; CD138^+^CD44^+^). FMO controls are shown in the right two columns.

Supplementary Figure 5 Comparison of CD103^+^ and CD103^-^ B cells in NLRP6-sufficient and -deficient mice. CD103^+^ and CD103^-^ B cells from 12-16-week-old NLRP6+/+NOD or NLRP6-/-NOD mice were investigated for changes to B cell subsets. CD19^+^ B cells were gated from live, single CD19^+^TCRβ^-^CD11b^-^CD11c^-^ cells prior to gating on CD103^+^ or CD103^-^ populations and subsequent gating on the specific surface receptor. Summarized proportions of CD1d^hi^CD5^hi^ **(A, B)**, splenic CD21^+^CD23^+^ c-c, e), splenic CD21^+^CD23^-^ **(D, F)**, splenic CD21^hi^CD24^hi^ **(G, I)**, splenic CD21^int^CD24^int^ (h, j) and CD138^+^CD44^+^ **(K, L)** B cells. Abbreviations include pancreatic lymph nodes (PLN), mesenteric lymph nodes (MLN) and Peyer’s patches (PP). Data were pooled from 2 independent experiments (n=6), with lines indicating the median value. Data were assessed for significance using a Student’s T-test.

Supplementary Figure 6 Comparisons of CD103^-^ B cell populations from NLRP6-sufficient and -deficient mice. CD103^-^ B cells from 12-16-week-old NLRP6+/+NOD or NLRP6-/-NOD mice were investigated for changes in B cell populations. CD19^+^ B cells were gated from live, single CD19^+^TCRβ^-^CD11b^-^CD11c^-^ cells prior to gating on CD103^+^ or CD103^-^ populations and subsequent gating on the specific populations. Summarized proportions of CD1d^hi^CD5^hi^ **(A)**, splenic CD21^+^CD23^+^ **(B)**, splenic CD21^+^CD23^-^ **(C)**, splenic CD21^hi^CD24^hi^ **(D)**, splenic CD21^int^CD24^int^ **(E)** and CD138^+^CD44^+^ **(F)** B cells. Abbreviations include pancreatic lymph nodes (PLN), mesenteric lymph nodes (MLN) and Peyer’s patches (PP). Data were pooled from 2 independent experiments (n=6), with lines indicating the median value. Data were assessed for significance using a Student’s T-test.

Supplementary Figure 7 Cytokine-secreting CD103^+^ B cells in NLRP6-sufficient and -deficient mice. Cytokine-secreting CD103^+^ B cells from 12-16-week-old NLRP6+/+NOD and NLRP6-/-NOD mice were investigated following brief 4-hour PMA and Ionomycin stimulation in the presence of Golgi Plug. Representative flow cytometric gating of intracellular cytokine data presented in . Flow cytometric plots show FMO/Isotype controls, NLRP6+/+NOD and NLRP6-/-NOD data from left to right, with IL-10, IL-17a, TGFβ1 and IL-21 from top to bottom. All were gated from isotype controls except IL17a, where a FMO control was used.

Supplementary Figure 8 Non-significant cytokine-secreting CD103^+^ B cells between NLRP6-sufficient and -deficient mice. Cytokine-secreting CD103^+^ B cells from 12-16-week-old NLRP6+/+NOD and NLRP6-/-NOD mice were investigated following brief 4-hour PMA and Ionomycin stimulation in the presence of Golgi Plug. The proportion of IL-4- **(A)**, IL-6- **(B)**, IFNγ- **(C)**, TNFα- **(D)** secreting B cells were gated from live, single CD19^+^TCRβ^-^CD11b^-^CD11c^-^CD103^+^ cells prior to gating on the specific cytokine. Abbreviations include pancreatic lymph nodes (PLN), mesenteric lymph nodes (MLN) and Peyer’s patches (PP). Data were pooled from 2 independent experiments (n=6), with lines indicating the median value. Data were assessed for significance using a Student’s T-test

Supplementary Figure 9 Comparison of cytokine-secreting B cells between CD103^+^ and CD103^-^ B cells in NLRP6-sufficient and -deficient mice. Cytokine-secreting CD103^+^ and CD103^-^ B cells from 12-16-week-old NLRP6+/+NOD and NLRP6-/-NOD mice were investigated following brief 4-hour PMA and Ionomycin stimulation in the presence of Golgi Plug. The proportion of IL-4- **(A)**, IL-6- **(B)**, IL-10- **(C)**, IL-17a- **(D)**, IL-21- **(E)**, IFNγ- **(F)**, TGFβ1- **(G)**, TNFα- **(H)** secreting B cells were gated from live, single CD19^+^TCRβ^-^CD11b^-^CD11c^-^CD103^+^ cells prior to gating on the specific cytokine. NLRP6+/+NOD data are shown on the left hand-side column with NLRP6-/-NOD data shown on the right hand-side column. Abbreviations include pancreatic lymph nodes (PLN), mesenteric lymph nodes (MLN) and Peyer’s patches (PP). Data were pooled from 2 independent experiments (n=6), with lines indicating the median value. Data were assessed for significance using a Student’s T-test.

Supplementary Figure 10 MHC expression and costimulatory marker proportions of CD103^+^ and CD103^-^ B cells in NLRP6-sufficient and -deficient mice. Splenic CD103^+^ and CD103^-^ B cells from 12-16-week-old NLRP6+/+NOD and NLRP6-/-NOD mice were investigated for MHC expression and costimulatory markers. The median fluorescence intensity of MHCII (H2-IA^g7^; **(A)**) and MHCI (H2-K^d^) **(B)**. The proportions of CD40^+^ **(C)**, CD80^+^ **(D)** and CD86^+^ **(E)** B cells. B cells were gated from live, single CD19^+^TCRβ^-^CD11b^-^CD11c^-^CD103^+^ cells prior to gating on the specific marker. Data were pooled from 2 independent experiments (n=6), with lines indicating the median value. Data were assessed for significance using a Student’s T-test.

Supplementary Figure 11 Identification of pathways involved in inducing CD103^+^ B cells. FACS-sorted CD103^+^ and CD103^-^ B cells from 12-16-week-old NLRP6+/+NOD and NLRP6-/-NOD mice were investigated for gene expression changes by RNA microarray (n=2/group from two different experiments). **(A)** Heat map of normalized gene expression data showing the significant differences between NLRP6-sufficient CD103^-^ vs CD103^+^ B cells and NLRP6-deficient CD103^-^ vs CD103^+^ B cells. High gene expression is shown in red, while green indicates low gene expression. Data are organized by genes increased or decreased in the order of significance. Data is organized into 3 comparisons NLRP6+/+ CD103^+^ vs CD103^-^ (top of heatmap), NLRP6+/+ and NLRP6-/- CD103^+^ vs CD103- (shared differences; middle of heatmap) and NLRP6-/- CD103^+^ vs CD103- (bottom of heatmap). A full list of genes from this heat map with results is presented in ESM Table 2. **(B, C)** Upstream analysis using IPA software showing the activation z-score of the top 20 regulators of NLRP6+/+ vs NLRP6-/- CD103+ B cells **(B)** or NLRP6+/+ CD103^-^ vs CD103^+^ B cells **(C)**. Data are organized from top to bottom in the order of significance (most significant at top).

Supplementary Figure 12 A higher proportion of NLRP6-deficient CD103^+^ B cells express TLR4 than NLRP6-sufficient CD103^+^ B cells. CD103^+^ and CD103^-^ B cells from 12-16-week-old NLRP6+/+NOD or NLRP6-/-NOD mice were investigated for changes in TLR4. CD19^+^ B cells were gated from live, single CD19^+^TCRβ^-^CD11b^-^CD11c^-^ cells prior to gating on CD103 by TLR4. **(A)** Representative gating of CD103^+^TLR4^+^ B cells and CD103^-^TLR4^+^ B cells. **(B, C)** Summarized proportions of CD103^+^TLR4^+^ B cells **(B)** and CD103^-^TLR4^+^ B cells **(C)**. (d-e) Median fluorescence intensity (MFI) of TLR4 is also shown for CD103^+^TLR4^+^ B cells **(D)** and CD103^-^TLR4^+^ B cells **(E)**. Data shown are from 1 of 2 independent experiments (n=4-5/experiment), with lines indicating the mean and SD. Data were assessed for significance using a Student’s T-test.

Supplementary Table 1 qPCR Primer Sequences.

Supplementary Table 2 Gene list and results from **Supplementary Figure 11A**.
