# Supplementary material for: NLRP6 deficiency expands a novel CD103+ B cell population that confers immune tolerance in NOD mice
Source: Front Immunol. 2023 Feb 23;14:1147925. doi: 10.3389/fimmu.2023.1147925 (PMC9995752; doi:10.3389/fimmu.2023.1147925)
Supplement: Supplementary Figure 1 — NLRP6 deficiency alters B cell populations. Pancreas infiltration and B cells were investigated from intestinal and peripheral tissues of 12-16-week-old NLRP6+/+NOD and NLRP6-/-NOD mice by flow cytometry. (A) Histological summary of islet immune cell infiltration (n=5 mice; islet totals 110-140). (B) The proportion and number of B cells in peripheral tissues, gated from single, live CD45+TCRβ-CD11b-CD11c- cells prior to gating on CD19+ cells. (C) The proportion of CD103+ B cells from the pancreas. (D-F) The median fluorescent intensity (MFI) of CD103 on B cells from the intestinal epithelial layer (D), lamina propria (E) and peripheral tissues (F). Abbreviations include pancreatic lymph nodes (PLN), mesenteric lymph nodes (MLN) and Peyer’s patches (PP). Data were pooled from 2 independent experiments (n=6), with lines indicating the median value. Data were assessed for significance using a Chi-square test (A) or a Student’s T-test. [file DataSheet_1.docx]

Supplementary Material

**NLRP6 deficiency expands a novel CD103^+^ B cell population that confers immune tolerance in NOD mice**

**James A. Pearson^1,3*^, Jian Peng^1^, Juan Huang^1^, Xiaoqing Yu^2^, Ningwen Tai^1^, Youjia Hu^1^, Sha Sha^1^, Richard A. Flavell^4,5^, Hongyu Zhao^2^, F. Susan Wong^3^ and Li Wen^1*^**

*** Correspondence:**
Dr James Alexander Pearson: [pearsonj1@cardiff.ac.uk](mailto:pearsonj1@cardiff.ac.uk) or Dr Li Wen: [li.wen@yale.edu](mailto:li.wen@yale.edu)

## Supplementary Figures

*Supplementary Figure 1. NLRP6 deficiency alters B cell populations.*

Pancreas infiltration and B cells were investigated from intestinal and peripheral lymphoid tissues of 12-16-week-old NLRP6+/+NOD and NLRP6-/-NOD mice by flow cytometry. (a) Histological summary of islet immune cell infiltration (n=5 mice; n=110-140 islets were evaluated). (b) The proportion and number of B cells in peripheral tissues, gated from single, live CD45^+^TCRβ^-^CD11b^-^CD11c^-^ cells prior to gating on CD19^+^ cells. (c) The proportion of CD103^+^ B cells from the pancreas. (d-f) The median fluorescent intensity (MFI) of CD103 on B cells from the intestinal epithelial layer (d), lamina propria (e) and peripheral lymphoid tissues (f). Abbreviations include pancreatic lymph nodes (PLN), mesenteric lymph nodes (MLN) and Peyer’s patches (PP). Data were pooled from 2 independent experiments (n=6), with lines indicating the median value. Data were assessed for significance using a Chi-square test (A) or a two-tailed Student’s T-test.

**

*Supplementary Figure 2. NLRP6 deficiency minimally alters other antigen-presenting cell populations.*

Antigen-presenting cells were investigated from intestinal and peripheral tissues of 12-16-week-old NLRP6+/+NOD and NLRP6-/-NOD mice by flow cytometry. All cells were gated from single, live CD45^+^TCRβ^-^CD19^-^ cells prior to gating on either CD11b^+^CD11c^+^, CD11b^-^CD11c^+^ or CD11b^+^CD11c^-^ cells. (a-c) The proportion of CD11b^+^CD11c^+^ cells were investigated from the intestinal epithelial layer (a), lamina propria (b) and peripheral lymphoid tissues (c). (d-f) The proportion of CD11c^+^(CD11b^-^) cells were investigated from the intestinal epithelial layer (d), lamina propria (e) and peripheral lymphoid tissues (f). (g-i) The proportion of CD11b^+^(CD11c^-^) cells were investigated from the intestinal epithelial layer (g), lamina propria (h) and peripheral lymphoid tissues (i). (j-l) The proportion of CD103^+^CD11c^+^(CD11b^-^) cells were investigated from the intestinal epithelial layer (j), lamina propria (k) and peripheral lymphoid tissues (l). Cells were gated from CD11c^+^CD11b^-^ cells prior to gating on CD103^+^ cells. Abbreviations include pancreatic lymph nodes (PLN), mesenteric lymph nodes (MLN) and Peyer’s patches (PP). Data were pooled from 2 independent experiments (n=6), with lines indicating the median value. Data were assessed for significance using a two-tailed Student’s T-test.

*Supplementary Figure 3. NLRP6 deficiency does not alter T cell proportion*

CD4^+^ and CD8^+^ T cells were investigated from the intestinal and peripheral lymphoid tissues of 12-16-week-old NLRP6+/+NOD and NLRP6-/-NOD mice by flow cytometry. (a-c) The proportion of CD4^+^ T cells were investigated from the intestinal epithelial layer (a), lamina propria (b) and peripheral lymphoid tissues (c). Intestinal cells were gated from single, live CD45^+^ cells prior to gating on TCRbeta^+^CD4^+^, while peripheral lymphoid cells were gated from live CD45^+^ TCRbeta^+^CD19^-^ cells prior to gating on CD4. (d-f) The proportion of CD8^+^ T cells were investigated from the intestinal epithelial layer (d), lamina propria (e) and peripheral lymphoid tissues (f). Cells were gated as above for CD4^+^ T cells, but on CD8^+^ T cells. (g-i) The proportion of CD4^+^CD25^+^FoxP3^+^ Tregs were investigated from the intestinal epithelial layer (g), lamina propria (h) and peripheral lymphoid tissues (i). Cells were gated as above for CD4^+^ T cells prior to gating on CD25^+^FoxP3^+^ cells. Abbreviations include pancreatic lymph nodes (PLN), mesenteric lymph nodes (MLN) and Peyer’s patches (PP). Data were pooled from 2 independent experiments (n=6), with lines indicating the median value. Data were assessed for significance using a two-tailed Student’s T-test.


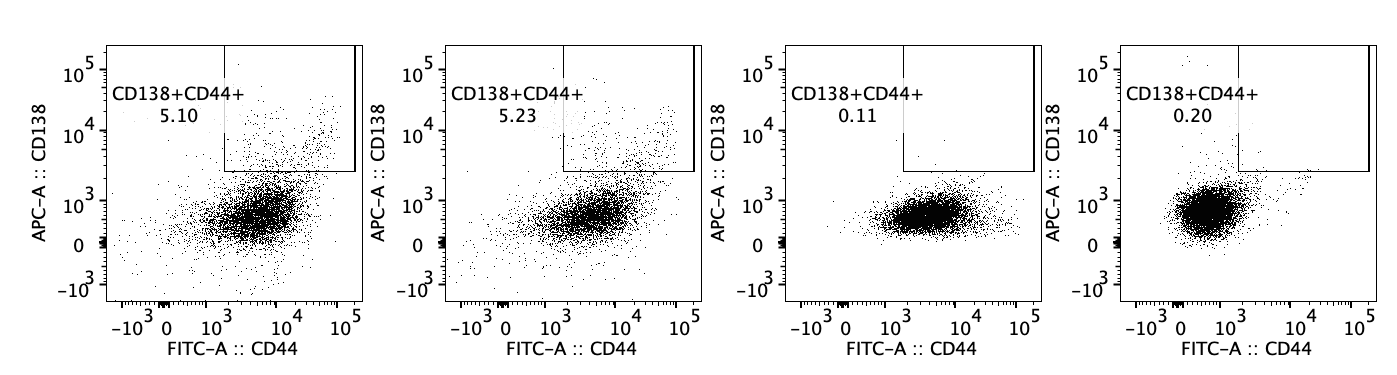

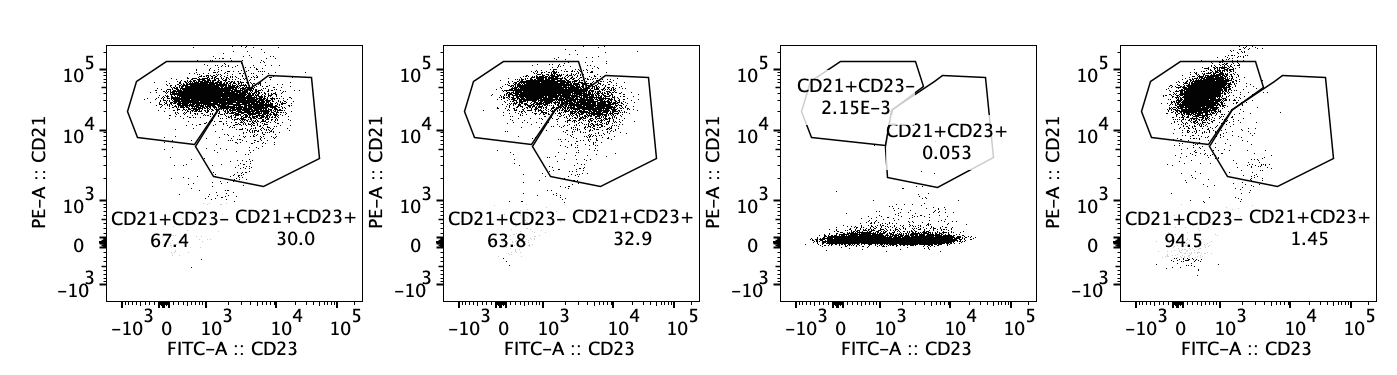

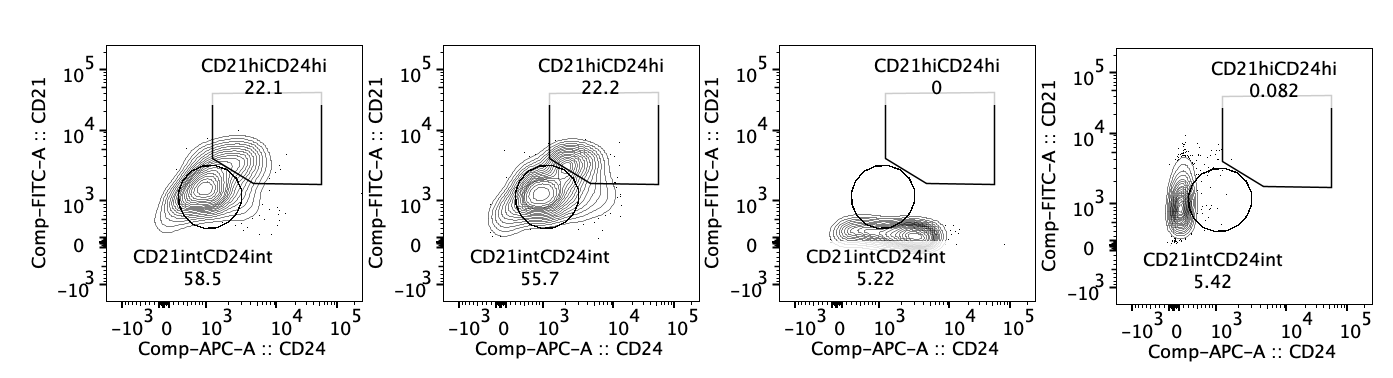

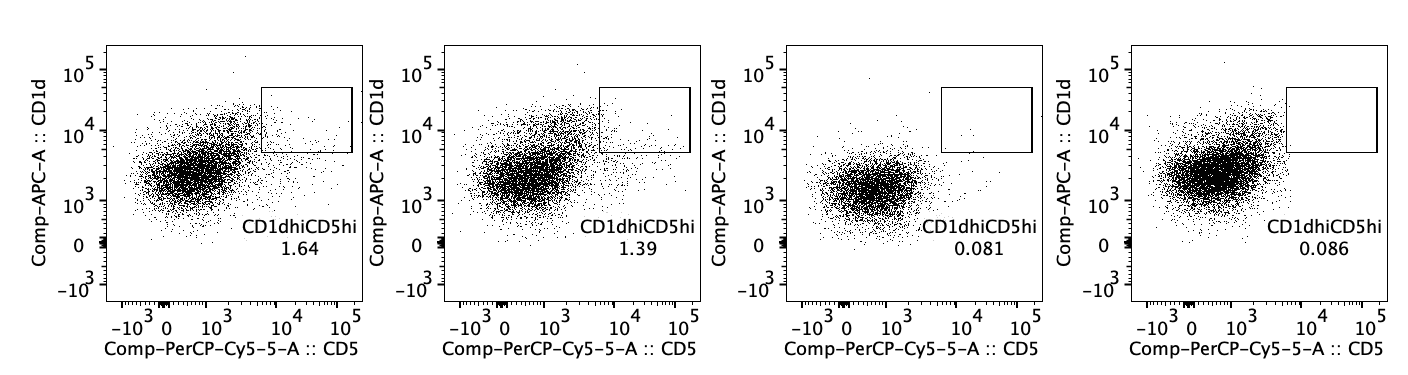


A

B

C

D

NLRP6+/+NOD

NLRP6-/-NOD

FMOs

*Supplementary Figure 4. Gating strategy of B cell subsets*

CD19^+^ B cells were gated from live, single CD19^+^TCRβ^-^CD11b^-^CD11c^-^ cells prior to gating on either CD103+ or CD103- populations. Cells were then investigated for regulatory B10 cells (a; CD1d^hi^CD5^hi^), regulatory marginal zone B cells (b-c; CD21^+^CD23^+/-^, CD21^hi^CD24^hi^ and CD21^Int^CD24^int^) and plasmablast Breg populations (d; CD138^+^CD44^+^). Fluorescence Minus One (FMO) controls are shown in the right two columns.

**

*Supplementary Figure 5. Comparison of CD103^+^ and CD103^-^ B cells in NLRP6-sufficient and -deficient mice*

CD103^+^ and CD103^-^ B cells from 12-16-week-old NLRP6+/+NOD or NLRP6-/-NOD mice were investigated for changes to B cell subsets. CD19^+^ B cells were gated from live, single CD19^+^TCRβ^-^CD11b^-^CD11c^-^ cells prior to gating on CD103^+^ or CD103^-^ populations and subsequent gating on the specific surface receptor. Summarized proportions of CD1d^hi^CD5^hi^ (a-b), splenic CD21^+^CD23^+^ (c,e), splenic CD21^+^CD23^-^ (d, f), splenic CD21^hi^CD24^hi^ (g, i), splenic CD21^int^CD24^int^ (h, j) and CD138^+^CD44^+^ (k-l) B cells. Abbreviations include pancreatic lymph nodes (PLN), mesenteric lymph nodes (MLN) and Peyer’s patches (PP). Data were pooled from 2 independent experiments (n=6), with lines indicating the median value. Data were assessed for significance using a two-tailed Student’s T-test.

*Supplementary Figure 6. Comparisons of CD103^-^ B cell populations from NLRP6-sufficient and -deficient mice*

CD103^-^ B cells from 12-16-week-old NLRP6+/+NOD or NLRP6-/-NOD mice were investigated for changes in B cell populations. CD19^+^ B cells were gated from live, single CD19^+^TCRβ^-^CD11b^-^CD11c^-^ cells prior to gating on CD103^+^ or CD103^-^ populations and subsequent gating on the specific populations. Summarized proportions of CD1d^hi^CD5^hi^ (a), splenic CD21^+^CD23^-^ (b), splenic CD21^+^CD23^+^ (c), splenic CD21^hi^CD24^hi^ (d), splenic CD21^int^CD24^int^ (e) and CD44^+^CD138^+^ (f) B cells. Abbreviations include pancreatic lymph nodes (PLN), mesenteric lymph nodes (MLN) and Peyer’s patches (PP). Data were pooled from 2 independent experiments (n=6), with lines indicating the median value. Data were assessed for significance using a two-tailed Student’s T-test


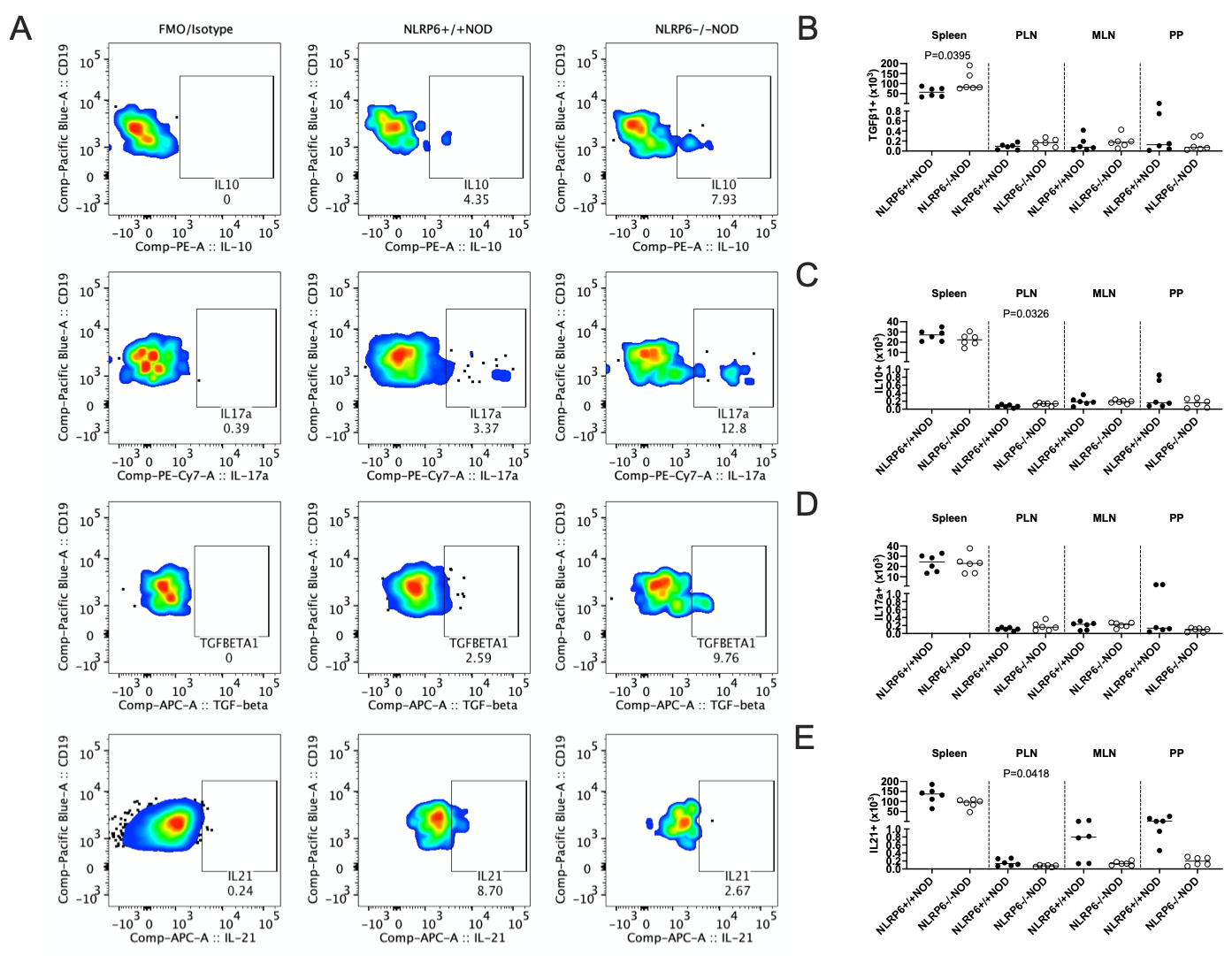


*Supplementary Figure 7. Cytokine-secreting CD103^+^ B cells in NLRP6-sufficient and -deficient mice with marked differences*

Cytokine-secreting CD103^+^ B cells from 12-16-week-old NLRP6+/+NOD and NLRP6-/-NOD mice were investigated following brief 4-hour PMA and Ionomycin stimulation in the presence of Golgi Plug. Representative flow cytometric gating of intracellular cytokine data presented in Fig. 2h-k. Flow cytometric plots show FMO/Isotype controls, NLRP6+/+NOD and NLRP6-/-NOD data from left to right, with IL-10, IL-17a, TGFβ1 and IL-21 from top to bottom. All were gated from isotype controls except IL17a, where a FMO control was used.

*Supplementary Figure 8. Non-significant cytokine-secreting CD103^+^ B cells between NLRP6-sufficient and -deficient mice*

Cytokine-secreting CD103^+^ B cells from 12-16-week-old NLRP6+/+NOD and NLRP6-/-NOD mice were investigated by flow cytometry following brief 4-hour PMA and Ionomycin stimulation in the presence of Golgi Plug. The proportion of IL-4- (a), IL-6- (b), IFNγ- (c), TNFα- (d) secreting B cells were gated from live, single CD19^+^TCRβ^-^CD11b^-^CD11c^-^CD103^+^ cells prior to gating on the specific cytokine. Abbreviations include pancreatic lymph nodes (PLN), mesenteric lymph nodes (MLN) and Peyer’s patches (PP). Data were pooled from 2 independent experiments (n=6), with lines indicating the median value. Data were assessed for significance using a two-tailed Student’s T-test

*Supplementary Figure 9. Comparison of cytokine-secreting B cells between CD103^+^ and CD103^-^ B cells in NLRP6-sufficient and -deficient mice*

Cytokine-secreting CD103^+^ and CD103^-^ B cells from 12-16-week-old NLRP6+/+NOD and NLRP6-/-NOD mice were investigated by flow cytometry following brief 4-hour PMA and Ionomycin stimulation in the presence of Golgi Plug. The proportion of IL-4- (a), IL-6- (b), IL-10- (c), IL-17a- (d), IL-21- (e), IFNγ- (f), TGFβ1- (g), TNFα- (h) secreting B cells were gated from live, single CD19^+^TCRβ^-^CD11b^-^CD11c^-^CD103^+^ cells prior to gating on the specific cytokine. NLRP6+/+NOD data are shown on the left hand-side column with NLRP6-/-NOD data shown on the right hand-side column. Abbreviations include pancreatic lymph nodes (PLN), mesenteric lymph nodes (MLN) and Peyer’s patches (PP). Data were pooled from 2 independent experiments (n=6), with lines indicating the median value. Data were assessed for significance using a two-tailed Student’s T-test.

*Supplementary Figure 10. MHC expression and costimulatory marker proportions of CD103^+^ and CD103^-^ B cells in NLRP6-sufficient and -deficient mice*

Splenic CD103^+^ and CD103^-^ B cells from 12-16-week-old NLRP6+/+NOD and NLRP6-/-NOD mice were investigated for MHC expression and costimulatory markers. The median fluorescence intensity (MFI) of MHCII (H2-IA^g7^; (a)) and MHCI (H2-K^d^) (b). The proportions of CD40^+^ (c), CD80^+^ (d) and CD86^+^ (e) B cells. B cells were gated from live, single CD19^+^TCRβ^-^CD11b^-^CD11c^-^CD103^+^ cells prior to gating on the specific marker. Data were pooled from 2 independent experiments (n=6), with lines indicating the median value. Data were assessed for significance using a two-tailed Student’s T-test.

*Supplementary Figure 11. Identification of pathways involved in inducing CD103^+^ B cells*

FACS-sorted CD103^+^ and CD103^-^ B cells from 12-16-week-old NLRP6+/+NOD and NLRP6-/-NOD mice were investigated for gene expression changes by RNA microarray (n=2/group from two different experiments). (a) Heat map of normalized gene expression data showing the significant differences between NLRP6-sufficient CD103^-^ vs CD103^+^ B cells and NLRP6-deficient CD103^-^ vs CD103^+^ B cells. Genes with high expression are shown in red, while green indicates the genes with low expression. Data are organized by genes increased or decreased in the order of significance. Data is organized into 3 comparisons NLRP6+/+ CD103^+^ vs CD103^-^ (top of heatmap), NLRP6+/+ and NLRP6-/- CD103^+^ vs CD103- (shared differences; middle of heatmap) and NLRP6-/- CD103^+^ vs CD103- (bottom of heatmap). A full list of genes from this heat map with results is presented in Supplementary Table 2. (b-c) Upstream analysis using IPA software showing the activation z-score of the top 20 regulators of NLRP6+/+ vs NLRP6-/- CD103+ B cells (b) or NLRP6+/+ CD103^-^ vs CD103^+^ B cells (c). Data are organized from top to bottom in the order of significance (most significant at top).

***
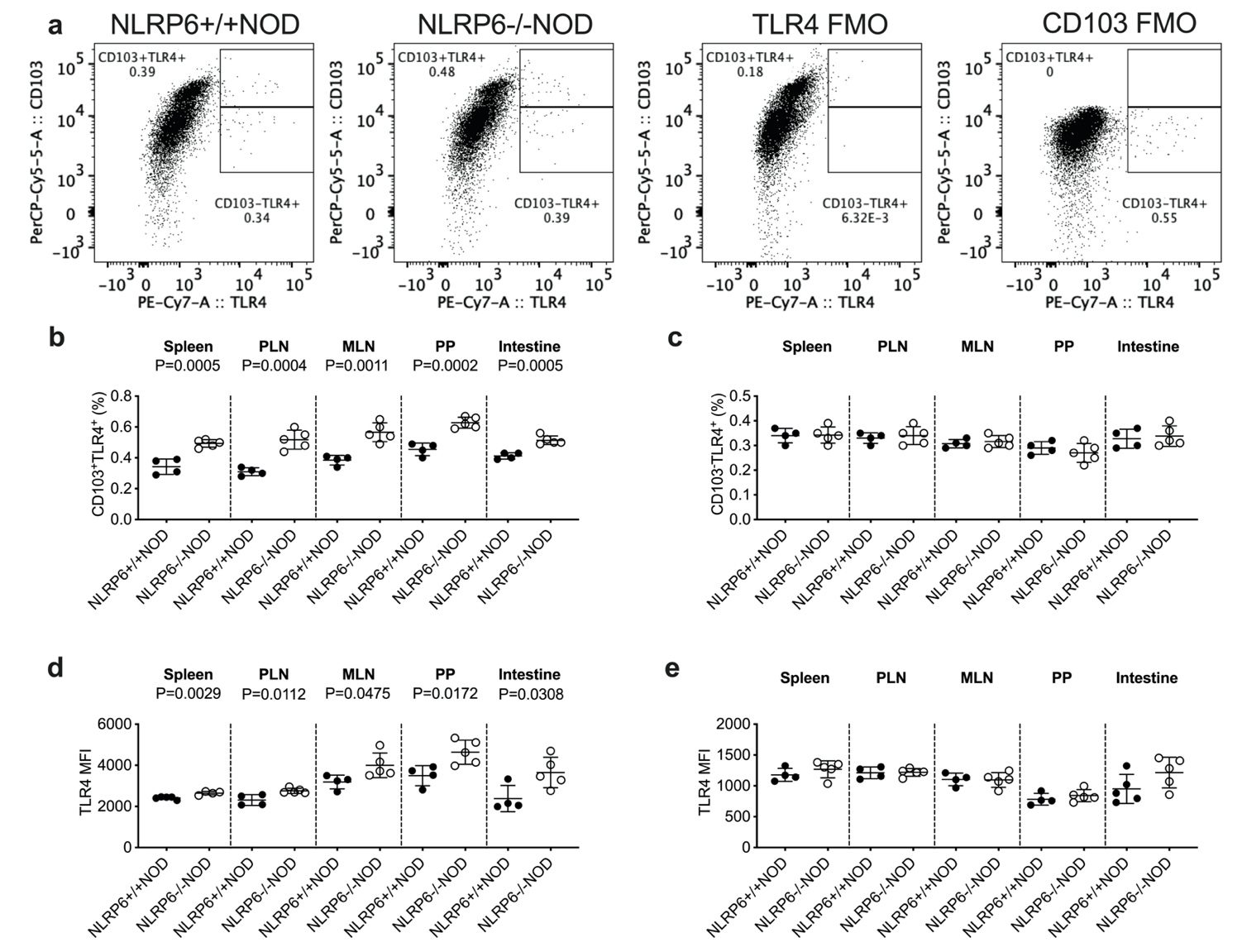
***

*Supplementary Figure 12. A higher proportion of NLRP6-deficient CD103^+^ B cells express TLR4 than NLRP6-sufficient CD103^+^ B cells*

CD103^+^ and CD103^-^ B cells from 12-16-week-old NLRP6+/+NOD or NLRP6-/-NOD mice were investigated for changes in TLR4. CD19^+^ B cells were gated from live, single CD19^+^TCRβ^-^CD11b^-^CD11c^-^ cells prior to gating on CD103 by TLR4. (a) Representative gating of CD103^+^TLR4^+^ B cells and CD103^-^TLR4^+^ B cells. (b-c) Summarized proportions of CD103^+^TLR4^+^ B cells (b) and CD103^-^TLR4^+^ B cells (c). (d-e) Median fluorescence intensity (MFI) of TLR4 is also shown to be higher in CD103^+^TLR4^+^ B cells in NLRP6-/-NOD mice (d) but not different in CD103^-^TLR4^+^ B cells (e). Data shown are from 1 of 2 independent experiments (n=4-5/experiment), with lines indicating the mean and SD. Data were assessed for significance using a two-tailed Student’s T-test.

Supplementary Table 1 – qPCR Primer Sequences

| **Gene** | **Forward (5’-3’)** | **Reverse (5’-3’)** |
| --- | --- | --- |
| ***Tlr4*** | TGTCATCAGGGACTTTGCTG | GGACTCTGATCATGGCACTG |
| ***Aldh1a1*** | ATGGTTTAGCAGCAGGACTCTTC | CCAGACATCTTGAATCCACCGAA |
| ***Aldh1a2*** | GACTTGTAGCAGCTGTCTTCACT | CACCCATTTCTCTCCCATTTCC |
| ***Aldh3a1*** | TAGGTGCTTGGAACTACCCATT | CCATAACGATCTTCCCTACAGC |
| ***Il-10*** | TGTCCAGCTGATCCTTCATTTG | ACCTGCCTAACATGCTTCGAG |
| ***Tgfβ1*** | GTGGTATACTGAGACACCTTGG | CCTTAGTTTGGACACGGATCTGG |
| ***Nlrp6*** | AGTCCTGACCACTCTGGACCTC | GACTGAGGGTCTTTAGGGAGCA |
| ***Reg3β*** | CTGCCTTAGACCGTGCTTTC | CCCTTGTCCATGATGCTCTT |
| ***Reg3γ*** | TTCCTGTCCTCCATGATCAAAA | CATCCACCTCTGTTGGGTTCA |
| ***Crp-ductin*** | TGAACCGTGTGACAGTGGTCTTCA | TCTCCTTGTCACACTGCCATCTGT |
| ***Relmb*** | AGCTCTCAGTCGTCAAGAGCCTAA | CACAAGCACATCCAGTGACAACCA |
| ***Defcr*** | ATCATCCAGGTGATTCCCAGCCAT | TTCCGGGTCTCCAAAGGAAACAGA |
| ***Il-18*** | GCCAAGCAAGAAAGTGTCCT | CAGTGAACCCCAGACCAGAC |
| ***Muc2*** | GCTGACGAGTGGTTGGTGAATG | GATGAGGTGGCAGACAGGAGAC |
| ***Zonulin1*** | CACCGGAGTGATGGTTTTCT | CCACCTCTGTCCAGCTCTTC |
| ***Gapdh*** | TGACATCAAGAAGGTGGTGAAG | TGCTGTAGCCGTATTCATTGTC |

Supplementary Table 2 – Gene list and results from Supplementary Fig. 11A

| **Gene ID** | **NLRP6+/+ CD103- 1** | **NLRP6+/+ CD103- 2** | **NLRP6-/- CD103- 1** | **NLRP6-/- CD103- 2** | **NLRP6+/+ CD103+ 1** | **NLRP6+/+ CD103+ 2** | **NLRP6-/- CD103+ 1** | **NLRP6-/- CD103+ 2** |
| --- | --- | --- | --- | --- | --- | --- | --- | --- |
| ***Prr11*** | 0.0822 | 0.0802 | 0.0935 | 0.0964 | 0.2137 | 0.2229 | 0.1080 | 0.1030 |
| ***E2f8*** | 0.0687 | 0.0685 | 0.0814 | 0.0755 | 0.2436 | 0.2677 | 0.0925 | 0.1021 |
| ***Dtl*** | 0.0894 | 0.0942 | 0.0911 | 0.0933 | 0.1931 | 0.2098 | 0.1144 | 0.1147 |
| ***S1pr2*** | 0.0824 | 0.0794 | 0.0953 | 0.0918 | 0.2217 | 0.2401 | 0.0889 | 0.1006 |
| ***Ighe*** | 0.0533 | 0.0457 | 0.0619 | 0.0493 | 0.3278 | 0.3215 | 0.0728 | 0.0677 |
| ***Casc5*** | 0.0763 | 0.0853 | 0.0962 | 0.0856 | 0.2251 | 0.2392 | 0.0957 | 0.0967 |
| ***Gm22792*** | 0.0767 | 0.0845 | 0.0918 | 0.0810 | 0.1956 | 0.1875 | 0.1427 | 0.1402 |
| ***Kif15*** | 0.0908 | 0.0901 | 0.1011 | 0.1074 | 0.1983 | 0.2173 | 0.0912 | 0.1038 |
| ***Top2a*** | 0.0733 | 0.0700 | 0.0865 | 0.0678 | 0.2434 | 0.2412 | 0.1056 | 0.1122 |
| ***Birc5*** | 0.0681 | 0.0611 | 0.1010 | 0.0781 | 0.2381 | 0.2394 | 0.1111 | 0.1031 |
| ***Cdc6*** | 0.1391 | 0.1158 | 0.1285 | 0.1165 | 0.1280 | 0.1359 | 0.1263 | 0.1099 |
| ***Gmnn*** | 0.0689 | 0.0764 | 0.0822 | 0.0881 | 0.1954 | 0.2269 | 0.1398 | 0.1222 |
| ***Cdca3*** | 0.0883 | 0.0871 | 0.1065 | 0.0893 | 0.1887 | 0.1905 | 0.1252 | 0.1243 |
| ***Cenpe*** | 0.0873 | 0.0922 | 0.1175 | 0.0977 | 0.1993 | 0.1957 | 0.1062 | 0.1042 |
| ***Tpx2*** | 0.0717 | 0.0784 | 0.0958 | 0.0812 | 0.2059 | 0.2212 | 0.1332 | 0.1126 |
| ***Plk1*** | 0.0614 | 0.0575 | 0.0818 | 0.0710 | 0.2490 | 0.2400 | 0.1391 | 0.1002 |
| ***Tmem176a*** | 0.0811 | 0.0897 | 0.0799 | 0.0862 | 0.1677 | 0.1850 | 0.1646 | 0.1458 |
| ***Espl1*** | 0.0913 | 0.0890 | 0.1138 | 0.1140 | 0.1733 | 0.1899 | 0.1048 | 0.1240 |
| ***Igkv17-127*** | 0.0775 | 0.0941 | 0.0742 | 0.0819 | 0.1853 | 0.1859 | 0.1495 | 0.1517 |
| ***Ccnb2*** | 0.0573 | 0.0668 | 0.0884 | 0.0696 | 0.2506 | 0.2335 | 0.1317 | 0.1022 |
| ***Ccna2*** | 0.0473 | 0.0424 | 0.0963 | 0.0603 | 0.2436 | 0.2653 | 0.1270 | 0.1178 |
| ***Cdca8*** | 0.0778 | 0.0886 | 0.0858 | 0.0783 | 0.1976 | 0.2044 | 0.1473 | 0.1203 |
| ***Cdk1*** | 0.0555 | 0.0612 | 0.0888 | 0.0839 | 0.1977 | 0.2795 | 0.1279 | 0.1055 |
| ***Uhrf1*** | 0.0954 | 0.0863 | 0.1052 | 0.1007 | 0.1855 | 0.2179 | 0.1121 | 0.0968 |
| ***H2afx*** | 0.0707 | 0.0906 | 0.1059 | 0.1115 | 0.1930 | 0.1832 | 0.1205 | 0.1246 |
| ***Cep55*** | 0.0888 | 0.0856 | 0.0997 | 0.0879 | 0.1860 | 0.2099 | 0.1103 | 0.1318 |
| ***2810417H13Rik*** | 0.0586 | 0.0703 | 0.1037 | 0.0732 | 0.2321 | 0.2264 | 0.1220 | 0.1136 |
| ***Ube2c*** | 0.0682 | 0.0707 | 0.0924 | 0.0694 | 0.2266 | 0.2376 | 0.1319 | 0.1031 |
| ***Eaf2*** | 0.0822 | 0.0718 | 0.0848 | 0.0669 | 0.2138 | 0.2062 | 0.1257 | 0.1485 |
| ***Ncapg*** | 0.0770 | 0.0647 | 0.0829 | 0.0762 | 0.2185 | 0.2454 | 0.1356 | 0.0998 |
| ***Mybl2*** | 0.0956 | 0.0853 | 0.1191 | 0.0978 | 0.1900 | 0.1807 | 0.1135 | 0.1180 |
| ***Fads1*** | 0.0733 | 0.0933 | 0.0970 | 0.0948 | 0.1665 | 0.1720 | 0.1555 | 0.1475 |
| ***Asns*** | 0.0883 | 0.0712 | 0.1069 | 0.0920 | 0.1775 | 0.1827 | 0.1316 | 0.1499 |
| ***Igh-VS107*** | 0.0415 | 0.0694 | 0.0600 | 0.0669 | 0.2735 | 0.3274 | 0.0920 | 0.0694 |
| ***Slc43a3*** | 0.0767 | 0.0935 | 0.0760 | 0.0921 | 0.1794 | 0.1823 | 0.1494 | 0.1506 |
| ***Nusap1*** | 0.0703 | 0.0802 | 0.1046 | 0.0767 | 0.1946 | 0.2220 | 0.1349 | 0.1167 |
| ***Mki67*** | 0.0266 | 0.0273 | 0.0690 | 0.0265 | 0.3224 | 0.3538 | 0.0919 | 0.0825 |
| ***Tyms*** | 0.0875 | 0.0931 | 0.0812 | 0.1041 | 0.1765 | 0.1924 | 0.1293 | 0.1359 |
| ***Esco2*** | 0.0861 | 0.0902 | 0.0962 | 0.0876 | 0.2005 | 0.1996 | 0.1382 | 0.1016 |
| ***Ighm*** | 0.0322 | 0.0399 | 0.0423 | 0.0328 | 0.0895 | 0.0849 | 0.3208 | 0.3575 |
| ***Rrm2*** | 0.0708 | 0.0753 | 0.1014 | 0.0673 | 0.2145 | 0.2028 | 0.1344 | 0.1334 |
| ***Emb*** | 0.0670 | 0.0892 | 0.1291 | 0.1200 | 0.1953 | 0.1847 | 0.1184 | 0.0963 |
| ***Hist1h2bk*** | 0.0501 | 0.0383 | 0.0919 | 0.0415 | 0.3042 | 0.3794 | 0.0469 | 0.0476 |
| ***Hbb-bt*** | 0.0678 | 0.0907 | 0.0924 | 0.0898 | 0.1927 | 0.1818 | 0.1264 | 0.1583 |
| ***Zdhhc2*** | 0.0813 | 0.0628 | 0.1070 | 0.1059 | 0.1851 | 0.1696 | 0.1644 | 0.1239 |
| ***Cdkn3*** | 0.0692 | 0.0937 | 0.1010 | 0.0960 | 0.1733 | 0.2091 | 0.1219 | 0.1359 |
| ***Spc24*** | 0.0679 | 0.0842 | 0.1013 | 0.0906 | 0.1803 | 0.2177 | 0.1481 | 0.1098 |
| ***LOC102642862*** | 0.0686 | 0.0872 | 0.0799 | 0.1009 | 0.1682 | 0.1676 | 0.1709 | 0.1567 |
| ***Ccnb1*** | 0.0775 | 0.0913 | 0.1064 | 0.0764 | 0.1916 | 0.2173 | 0.1235 | 0.1161 |
| ***Fut8*** | 0.0799 | 0.0937 | 0.1043 | 0.1022 | 0.1846 | 0.1795 | 0.1463 | 0.1096 |
| ***Gm19505*** | 0.0821 | 0.0960 | 0.1005 | 0.0896 | 0.1819 | 0.1894 | 0.1481 | 0.1124 |
| ***Kif23*** | 0.1018 | 0.0776 | 0.0972 | 0.0787 | 0.1936 | 0.2421 | 0.1016 | 0.1074 |
| ***Cdc20*** | 0.0786 | 0.1051 | 0.0811 | 0.0831 | 0.1845 | 0.2278 | 0.1270 | 0.1128 |
| ***Anxa2*** | 0.0749 | 0.0964 | 0.1122 | 0.0875 | 0.1798 | 0.1839 | 0.1391 | 0.1262 |
| ***Kif11*** | 0.0827 | 0.0787 | 0.1094 | 0.0757 | 0.2133 | 0.2387 | 0.1166 | 0.0849 |
| ***Igkv3-1*** | 0.0679 | 0.0989 | 0.0752 | 0.0906 | 0.1973 | 0.2048 | 0.1246 | 0.1407 |
| ***Ighv2-2*** | 0.0722 | 0.0967 | 0.0763 | 0.0958 | 0.1755 | 0.1857 | 0.1442 | 0.1535 |
| ***Ighv1-77*** | 0.0633 | 0.0979 | 0.0700 | 0.0818 | 0.2087 | 0.1979 | 0.1370 | 0.1432 |
| ***Hist1h2ab*** | 0.0692 | 0.0833 | 0.0983 | 0.1144 | 0.2061 | 0.1887 | 0.0962 | 0.1438 |
| ***LOC101056284*** | 0.0728 | 0.0895 | 0.0660 | 0.0952 | 0.1828 | 0.1849 | 0.1540 | 0.1549 |
| ***Plxnb2*** | 0.0721 | 0.0938 | 0.0843 | 0.1127 | 0.1790 | 0.1762 | 0.1461 | 0.1359 |
| ***4933402N22Rik*** | 0.0790 | 0.0713 | 0.1105 | 0.0741 | 0.1756 | 0.2112 | 0.1560 | 0.1224 |
| ***Igkv6-13*** | 0.0747 | 0.0982 | 0.0821 | 0.1060 | 0.1893 | 0.1664 | 0.1395 | 0.1439 |
| ***Pbk*** | 0.0940 | 0.0879 | 0.1161 | 0.0873 | 0.2086 | 0.1814 | 0.0978 | 0.1268 |
| ***Igh-VJ558*** | 0.0685 | 0.0819 | 0.0599 | 0.0991 | 0.2114 | 0.2106 | 0.1471 | 0.1214 |
| ***Basp1*** | 0.0997 | 0.0689 | 0.1171 | 0.0936 | 0.1841 | 0.1765 | 0.1332 | 0.1268 |
| ***Igkv4-73*** | 0.0684 | 0.0990 | 0.0706 | 0.0900 | 0.1817 | 0.1826 | 0.1486 | 0.1593 |
| ***Gm6460*** | 0.0795 | 0.0772 | 0.1195 | 0.0849 | 0.1582 | 0.1944 | 0.1588 | 0.1275 |
| ***Ighv1-37*** | 0.0740 | 0.1027 | 0.0745 | 0.0984 | 0.1829 | 0.1979 | 0.1295 | 0.1401 |
| ***Aicda*** | 0.1030 | 0.0804 | 0.1224 | 0.0817 | 0.1981 | 0.1989 | 0.1145 | 0.1009 |
| ***Gcsam*** | 0.0937 | 0.0877 | 0.1274 | 0.0775 | 0.1928 | 0.2288 | 0.0944 | 0.0977 |
| ***Igkv4-55*** | 0.0633 | 0.0968 | 0.0626 | 0.0907 | 0.2043 | 0.1847 | 0.1413 | 0.1564 |
| ***Ccne2*** | 0.0817 | 0.0911 | 0.1231 | 0.0857 | 0.1711 | 0.2108 | 0.1023 | 0.1342 |
| ***LOC102640775*** | 0.0717 | 0.1081 | 0.0891 | 0.1059 | 0.1707 | 0.1908 | 0.1265 | 0.1372 |
| ***Ighv6-6*** | 0.0578 | 0.0975 | 0.0441 | 0.0843 | 0.2521 | 0.2731 | 0.0934 | 0.0976 |
| ***Rgs13*** | 0.0833 | 0.0433 | 0.1231 | 0.0550 | 0.2827 | 0.2795 | 0.0688 | 0.0644 |
| ***Fam46c*** | 0.0913 | 0.0648 | 0.0992 | 0.0619 | 0.1756 | 0.2072 | 0.1570 | 0.1431 |
| ***Gm6460*** | 0.0743 | 0.0721 | 0.1053 | 0.0721 | 0.1552 | 0.2316 | 0.1682 | 0.1212 |
| ***Igkv4-57-1*** | 0.0690 | 0.1066 | 0.0703 | 0.0915 | 0.1846 | 0.1776 | 0.1447 | 0.1558 |
| ***Ighv1-43*** | 0.0694 | 0.1057 | 0.0662 | 0.0906 | 0.1839 | 0.1818 | 0.1509 | 0.1515 |
| ***Igkv4-68*** | 0.0679 | 0.1019 | 0.0615 | 0.0930 | 0.1934 | 0.1868 | 0.1457 | 0.1499 |
| ***H1f0*** | 0.0607 | 0.0987 | 0.0941 | 0.0990 | 0.1643 | 0.1512 | 0.1574 | 0.1746 |
| ***Igkv1-115*** | 0.0534 | 0.0778 | 0.0486 | 0.1004 | 0.2164 | 0.2212 | 0.1629 | 0.1193 |
| ***Igkv5-37*** | 0.0722 | 0.1096 | 0.0714 | 0.0883 | 0.1951 | 0.1668 | 0.1577 | 0.1390 |
| ***Igkv10-95*** | 0.0629 | 0.1068 | 0.0719 | 0.0907 | 0.1853 | 0.1798 | 0.1461 | 0.1564 |
| ***Igkv14-100*** | 0.0673 | 0.1016 | 0.0717 | 0.0917 | 0.1821 | 0.1767 | 0.1292 | 0.1797 |
| ***Ighv1-20*** | 0.0445 | 0.0941 | 0.0609 | 0.0874 | 0.2142 | 0.1845 | 0.1636 | 0.1508 |
| ***Il2rb*** | 0.0963 | 0.0827 | 0.1456 | 0.0844 | 0.1830 | 0.1955 | 0.1010 | 0.1115 |
| ***Igkv11-125*** | 0.0715 | 0.0874 | 0.0658 | 0.1130 | 0.2096 | 0.1629 | 0.1318 | 0.1580 |
| ***Hist1h2af*** | 0.0710 | 0.1051 | 0.0677 | 0.1131 | 0.2020 | 0.1961 | 0.1129 | 0.1323 |
| ***Gm20730*** | 0.0290 | 0.0767 | 0.0417 | 0.0808 | 0.2325 | 0.1998 | 0.1472 | 0.1923 |
| ***Igkv4-53*** | 0.0641 | 0.0937 | 0.0506 | 0.0902 | 0.2236 | 0.1897 | 0.1200 | 0.1680 |
| ***Mybl1*** | 0.0805 | 0.0577 | 0.1427 | 0.0512 | 0.2733 | 0.2605 | 0.0743 | 0.0597 |
| ***Ighv1-36*** | 0.0525 | 0.0916 | 0.0511 | 0.0982 | 0.1871 | 0.1908 | 0.1557 | 0.1730 |
| ***Hba-a2*** | 0.0790 | 0.0436 | 0.1262 | 0.0514 | 0.1980 | 0.1930 | 0.1641 | 0.1448 |
| ***Igkv6-29*** | 0.0635 | 0.1082 | 0.0738 | 0.1103 | 0.1775 | 0.1686 | 0.1428 | 0.1552 |
| ***Ighv8-8*** | 0.0654 | 0.1206 | 0.0729 | 0.0901 | 0.1859 | 0.1755 | 0.1348 | 0.1548 |
| ***Mir22*** | 0.1178 | 0.0617 | 0.1061 | 0.1000 | 0.1700 | 0.1723 | 0.1277 | 0.1444 |
| ***Igh-V10*** | 0.0651 | 0.0996 | 0.0661 | 0.1152 | 0.1651 | 0.1815 | 0.1310 | 0.1765 |
| ***Gm26202*** | 0.1585 | 0.1733 | 0.1751 | 0.1729 | 0.0725 | 0.0705 | 0.0847 | 0.0925 |
| ***mt-Tm*** | 0.1531 | 0.1599 | 0.1507 | 0.1661 | 0.0766 | 0.0689 | 0.1082 | 0.1164 |
| ***mt-Tw*** | 0.1819 | 0.2644 | 0.1265 | 0.1779 | 0.0461 | 0.0423 | 0.0790 | 0.0819 |
| ***Snord49b*** | 0.1497 | 0.1646 | 0.1646 | 0.1860 | 0.0707 | 0.0724 | 0.1060 | 0.0861 |
| ***Gm26205*** | 0.1390 | 0.1646 | 0.1494 | 0.1877 | 0.0658 | 0.0676 | 0.1136 | 0.1123 |
| ***Gm23130*** | 0.1723 | 0.1675 | 0.1486 | 0.1169 | 0.0772 | 0.0895 | 0.1151 | 0.1128 |
| ***Snord89*** | 0.1466 | 0.1799 | 0.1306 | 0.1746 | 0.0606 | 0.0741 | 0.1174 | 0.1162 |
| ***Snord42a*** | 0.1408 | 0.2084 | 0.1556 | 0.1585 | 0.0679 | 0.0491 | 0.1061 | 0.1136 |
| ***Gm26287*** | 0.1559 | 0.2769 | 0.1273 | 0.1290 | 0.0612 | 0.0647 | 0.0959 | 0.0892 |
| ***Picalm*** | 0.1871 | 0.1949 | 0.1266 | 0.1461 | 0.0724 | 0.0937 | 0.0737 | 0.1055 |
| ***Mir3068*** | 0.1466 | 0.2104 | 0.1566 | 0.1435 | 0.0635 | 0.0919 | 0.0969 | 0.0906 |
| ***Snord68*** | 0.1271 | 0.2136 | 0.1365 | 0.2041 | 0.0605 | 0.0602 | 0.0997 | 0.0984 |
| ***Gm22154*** | 0.1122 | 0.1564 | 0.1505 | 0.2046 | 0.0600 | 0.0648 | 0.1143 | 0.1372 |
| ***Snord70*** | 0.1288 | 0.2427 | 0.1399 | 0.2088 | 0.0566 | 0.0491 | 0.0733 | 0.1010 |
| ***Rny3*** | 0.1215 | 0.2191 | 0.1322 | 0.2652 | 0.0424 | 0.0477 | 0.0829 | 0.0891 |
| ***Snord17*** | 0.1131 | 0.1779 | 0.1707 | 0.2077 | 0.0663 | 0.0724 | 0.0903 | 0.1016 |
| ***Scarna9*** | 0.1249 | 0.2575 | 0.1466 | 0.1776 | 0.0704 | 0.0691 | 0.0629 | 0.0909 |
| ***Gm24616*** | 0.1025 | 0.2246 | 0.1449 | 0.2378 | 0.0510 | 0.0539 | 0.1076 | 0.0777 |
| ***Gm25788*** | 0.0908 | 0.1702 | 0.1475 | 0.2598 | 0.0458 | 0.0512 | 0.1045 | 0.1302 |
| ***Snord19*** | 0.1235 | 0.3539 | 0.1074 | 0.1624 | 0.0556 | 0.0737 | 0.0623 | 0.0613 |
| ***Gm25683*** | 0.0859 | 0.1771 | 0.1705 | 0.2845 | 0.0524 | 0.0471 | 0.0840 | 0.0985 |
| ***Ighv2-9*** | 0.0560 | 0.0491 | 0.0740 | 0.0710 | 0.1607 | 0.1777 | 0.1979 | 0.2137 |
| ***Ighg1*** | 0.0129 | 0.0178 | 0.0399 | 0.0283 | 0.2427 | 0.2564 | 0.2005 | 0.2014 |
| ***Kcnn4*** | 0.0691 | 0.0791 | 0.0752 | 0.0780 | 0.1770 | 0.1916 | 0.1709 | 0.1593 |
| ***Chst1*** | 0.0520 | 0.0634 | 0.0517 | 0.0573 | 0.2021 | 0.1922 | 0.1840 | 0.1973 |
| ***Ppapdc1b*** | 0.0718 | 0.0679 | 0.0884 | 0.0745 | 0.1871 | 0.1753 | 0.1681 | 0.1670 |
| ***Slpi*** | 0.0250 | 0.0350 | 0.0336 | 0.0441 | 0.2196 | 0.2334 | 0.2085 | 0.2007 |
| ***Tnfrsf17*** | 0.0621 | 0.0731 | 0.0696 | 0.0758 | 0.1656 | 0.1880 | 0.1796 | 0.1862 |
| ***Ly6c2*** | 0.0331 | 0.0305 | 0.0665 | 0.0431 | 0.2062 | 0.2022 | 0.2193 | 0.1990 |
| ***Cd68*** | 0.0531 | 0.0532 | 0.0492 | 0.0630 | 0.1410 | 0.1450 | 0.2532 | 0.2424 |
| ***Ighg2b*** | 0.0141 | 0.0265 | 0.0453 | 0.0325 | 0.2455 | 0.2458 | 0.2024 | 0.1878 |
| ***Ighg2c*** | 0.0241 | 0.0412 | 0.0505 | 0.0606 | 0.2189 | 0.2142 | 0.1986 | 0.1919 |
| ***Igh-VJ558*** | 0.0136 | 0.0263 | 0.0230 | 0.0338 | 0.2388 | 0.2474 | 0.2159 | 0.2013 |
| ***Tmem176b*** | 0.0770 | 0.0909 | 0.0765 | 0.0789 | 0.1731 | 0.1716 | 0.1776 | 0.1544 |
| ***C1qc*** | 0.0422 | 0.0525 | 0.0610 | 0.0752 | 0.1224 | 0.1333 | 0.2651 | 0.2482 |
| ***Pilra*** | 0.0555 | 0.0551 | 0.0607 | 0.0755 | 0.1115 | 0.1248 | 0.2623 | 0.2545 |
| ***Derl3*** | 0.0527 | 0.0715 | 0.0617 | 0.0779 | 0.1903 | 0.1917 | 0.1733 | 0.1809 |
| ***Kcnj10*** | 0.0616 | 0.0531 | 0.0625 | 0.0813 | 0.1518 | 0.1364 | 0.2289 | 0.2244 |
| ***Slc11a1*** | 0.0483 | 0.0474 | 0.0589 | 0.0774 | 0.1101 | 0.1298 | 0.2619 | 0.2661 |
| ***Ighv1-47*** | 0.0509 | 0.0686 | 0.0406 | 0.0583 | 0.2292 | 0.2302 | 0.1686 | 0.1535 |
| ***Ighv1-76*** | 0.0678 | 0.0865 | 0.0698 | 0.0859 | 0.1863 | 0.1837 | 0.1584 | 0.1617 |
| ***Ighv3-8*** | 0.0377 | 0.0564 | 0.0380 | 0.0566 | 0.2281 | 0.2098 | 0.1776 | 0.1959 |
| ***Hid1*** | 0.0718 | 0.0768 | 0.0775 | 0.0767 | 0.1649 | 0.1924 | 0.1456 | 0.1943 |
| ***Txndc5*** | 0.0720 | 0.0936 | 0.0733 | 0.0808 | 0.1666 | 0.1778 | 0.1654 | 0.1705 |
| ***Creb3l2*** | 0.0630 | 0.0717 | 0.0595 | 0.0828 | 0.1704 | 0.1812 | 0.1759 | 0.1956 |
| ***LOC238440*** | 0.0375 | 0.0633 | 0.0339 | 0.0527 | 0.2763 | 0.2695 | 0.1289 | 0.1379 |
| ***Ccr3*** | 0.0339 | 0.0356 | 0.0403 | 0.0580 | 0.1048 | 0.0977 | 0.3497 | 0.2799 |
| ***Ighv9-4*** | 0.0518 | 0.0764 | 0.0497 | 0.0705 | 0.2170 | 0.2350 | 0.1441 | 0.1556 |
| ***Ighv14-4*** | 0.0735 | 0.0948 | 0.0725 | 0.0856 | 0.1685 | 0.1700 | 0.1659 | 0.1693 |
| ***Slc7a8*** | 0.0408 | 0.0526 | 0.0445 | 0.0589 | 0.1493 | 0.1185 | 0.2834 | 0.2520 |
| ***Lrp1*** | 0.0524 | 0.0569 | 0.0574 | 0.0764 | 0.1245 | 0.1098 | 0.2714 | 0.2513 |
| ***Fcer1g*** | 0.0343 | 0.0376 | 0.0378 | 0.0645 | 0.1299 | 0.1166 | 0.2935 | 0.2857 |
| ***Pon3*** | 0.0303 | 0.0546 | 0.0261 | 0.0452 | 0.2390 | 0.2235 | 0.1953 | 0.1860 |
| ***LOC434035*** | 0.0482 | 0.0719 | 0.0514 | 0.0725 | 0.1806 | 0.1853 | 0.1797 | 0.2104 |
| ***Prg2*** | 0.0157 | 0.0382 | 0.0166 | 0.0287 | 0.1996 | 0.2239 | 0.2372 | 0.2401 |
| ***Mrc1*** | 0.0447 | 0.0539 | 0.0521 | 0.0773 | 0.1305 | 0.1171 | 0.2682 | 0.2561 |
| ***Emr4*** | 0.0302 | 0.0322 | 0.0284 | 0.0497 | 0.0989 | 0.1037 | 0.3026 | 0.3543 |
| ***Ighv1-53*** | 0.0651 | 0.0892 | 0.0483 | 0.0696 | 0.2006 | 0.2033 | 0.1658 | 0.1581 |
| ***Oosp1*** | 0.0584 | 0.0865 | 0.0751 | 0.0895 | 0.1612 | 0.1839 | 0.1677 | 0.1778 |
| ***Itgad*** | 0.0417 | 0.0522 | 0.0497 | 0.0884 | 0.1499 | 0.1670 | 0.2336 | 0.2176 |
| ***Cd63*** | 0.0492 | 0.0541 | 0.0611 | 0.1023 | 0.1447 | 0.1409 | 0.2307 | 0.2170 |
| ***Igkv5-45*** | 0.0570 | 0.0831 | 0.0518 | 0.0770 | 0.1957 | 0.1991 | 0.1696 | 0.1668 |
| ***Ighv4-1*** | 0.0324 | 0.0535 | 0.0277 | 0.0558 | 0.2252 | 0.2146 | 0.1875 | 0.2034 |
| ***Ighm*** | 0.0162 | 0.0454 | 0.0111 | 0.0221 | 0.2983 | 0.2840 | 0.1669 | 0.1559 |
| ***Fabp5*** | 0.0750 | 0.0404 | 0.0730 | 0.0817 | 0.1833 | 0.1835 | 0.1846 | 0.1784 |
| ***Igh-VX24*** | 0.0463 | 0.0749 | 0.0418 | 0.0625 | 0.1905 | 0.1894 | 0.1952 | 0.1993 |
| ***Igj*** | 0.0138 | 0.0389 | 0.0159 | 0.0341 | 0.2429 | 0.2364 | 0.2074 | 0.2106 |
| ***Ighv1-73*** | 0.0412 | 0.0971 | 0.0421 | 0.1014 | 0.2240 | 0.1713 | 0.1730 | 0.1499 |
| ***LOC435333*** | 0.0462 | 0.0741 | 0.0383 | 0.0702 | 0.2591 | 0.2275 | 0.1495 | 0.1351 |
| ***Abcc3*** | 0.0523 | 0.0573 | 0.0504 | 0.0729 | 0.1069 | 0.1182 | 0.2788 | 0.2634 |
| ***Igkv4-79*** | 0.0692 | 0.0950 | 0.0606 | 0.0821 | 0.1796 | 0.1774 | 0.1671 | 0.1690 |
| ***Fabp5*** | 0.0786 | 0.0416 | 0.0764 | 0.0829 | 0.1820 | 0.1808 | 0.1843 | 0.1734 |
| ***Igh-V3660*** | 0.0640 | 0.0910 | 0.0619 | 0.0863 | 0.1781 | 0.1888 | 0.1669 | 0.1630 |
| ***Igkv15-103*** | 0.0692 | 0.0989 | 0.0616 | 0.0834 | 0.1888 | 0.1933 | 0.1500 | 0.1548 |
| ***Igkv3-10*** | 0.0610 | 0.0930 | 0.0630 | 0.0834 | 0.1844 | 0.1906 | 0.1568 | 0.1679 |
| ***LOC101056278*** | 0.0736 | 0.0996 | 0.1061 | 0.0952 | 0.1939 | 0.1447 | 0.1457 | 0.1412 |
| ***Igh-V7183*** | 0.0457 | 0.0770 | 0.0617 | 0.0818 | 0.1679 | 0.1705 | 0.2004 | 0.1951 |
| ***Axl*** | 0.0225 | 0.0335 | 0.0314 | 0.0692 | 0.1334 | 0.1277 | 0.2881 | 0.2943 |
| ***Igkv4-86*** | 0.0451 | 0.0678 | 0.0340 | 0.0689 | 0.2378 | 0.2241 | 0.1574 | 0.1649 |
| ***Igkv7-33*** | 0.0147 | 0.0326 | 0.0074 | 0.0241 | 0.2525 | 0.2778 | 0.2032 | 0.1878 |
| ***Igkv2-137*** | 0.0450 | 0.0740 | 0.0430 | 0.0695 | 0.1966 | 0.1868 | 0.1945 | 0.1907 |
| ***Ighv1-22*** | 0.0733 | 0.1057 | 0.0657 | 0.0776 | 0.1907 | 0.1713 | 0.1571 | 0.1586 |
| ***Igkv3-5*** | 0.0585 | 0.0898 | 0.0537 | 0.0814 | 0.1992 | 0.1927 | 0.1586 | 0.1661 |
| ***Igkv2-112*** | 0.0577 | 0.0774 | 0.0528 | 0.0723 | 0.2225 | 0.1563 | 0.2057 | 0.1553 |
| ***Clec4n*** | 0.0324 | 0.0351 | 0.0364 | 0.0656 | 0.0869 | 0.0955 | 0.3148 | 0.3332 |
| ***C1qb*** | 0.0154 | 0.0195 | 0.0204 | 0.0668 | 0.1259 | 0.1176 | 0.3235 | 0.3108 |
| ***Trf*** | 0.0344 | 0.0399 | 0.0353 | 0.0759 | 0.1347 | 0.1251 | 0.2944 | 0.2603 |
| ***Adamdec1*** | 0.0466 | 0.0499 | 0.0488 | 0.0837 | 0.1210 | 0.1101 | 0.2623 | 0.2777 |
| ***Igh-VJ558*** | 0.0435 | 0.0844 | 0.0544 | 0.0718 | 0.1885 | 0.1996 | 0.1643 | 0.1935 |
| ***C1qa*** | 0.0194 | 0.0282 | 0.0292 | 0.0825 | 0.1288 | 0.1390 | 0.2973 | 0.2755 |
| ***LOC637260*** | 0.0695 | 0.0968 | 0.0686 | 0.0912 | 0.1925 | 0.1586 | 0.1668 | 0.1560 |
| ***Ighv14-1*** | 0.0610 | 0.0946 | 0.0624 | 0.0873 | 0.1994 | 0.1715 | 0.1575 | 0.1664 |
| ***Ighv6-3*** | 0.0430 | 0.0807 | 0.0454 | 0.0735 | 0.1955 | 0.2108 | 0.1677 | 0.1834 |
| ***Csf1r*** | 0.0281 | 0.0343 | 0.0339 | 0.0730 | 0.1060 | 0.1051 | 0.3248 | 0.2948 |
| ***Igh-VX24*** | 0.0702 | 0.0696 | 0.0576 | 0.0953 | 0.1638 | 0.1491 | 0.1862 | 0.2082 |
| ***Igkv4-56*** | 0.0553 | 0.0899 | 0.0561 | 0.0870 | 0.2110 | 0.1872 | 0.1455 | 0.1681 |
| ***Iglv2*** | 0.0489 | 0.0828 | 0.0518 | 0.0847 | 0.1908 | 0.1901 | 0.1778 | 0.1730 |
| ***Igkv3-7*** | 0.0375 | 0.0804 | 0.0341 | 0.0584 | 0.2161 | 0.2269 | 0.1667 | 0.1798 |
| ***Igh-VJ558*** | 0.0546 | 0.0936 | 0.0509 | 0.0775 | 0.2235 | 0.1867 | 0.1534 | 0.1598 |
| ***Fcna*** | 0.0246 | 0.0398 | 0.0324 | 0.0768 | 0.1366 | 0.1330 | 0.2886 | 0.2681 |
| ***Slc40a1*** | 0.0091 | 0.0170 | 0.0148 | 0.0629 | 0.1436 | 0.1122 | 0.3101 | 0.3303 |
| ***Ighv1-62-3*** | 0.0513 | 0.0799 | 0.0373 | 0.0732 | 0.2136 | 0.1982 | 0.1778 | 0.1686 |
| ***Gfra2*** | 0.0421 | 0.0448 | 0.0490 | 0.0697 | 0.1215 | 0.0943 | 0.3537 | 0.2250 |
| ***Ighv5-9-1*** | 0.0549 | 0.0950 | 0.0669 | 0.0875 | 0.1740 | 0.1767 | 0.1617 | 0.1833 |
| ***Treml4*** | 0.0316 | 0.0385 | 0.0320 | 0.0656 | 0.0942 | 0.1206 | 0.3007 | 0.3166 |
| ***Igkv9-123*** | 0.0376 | 0.0548 | 0.0193 | 0.0552 | 0.2326 | 0.2201 | 0.2042 | 0.1763 |
| ***Igkv4-50*** | 0.0633 | 0.0997 | 0.0588 | 0.0885 | 0.1915 | 0.1868 | 0.1507 | 0.1607 |
| ***Ighv1-56*** | 0.0449 | 0.0807 | 0.0327 | 0.0641 | 0.2282 | 0.2018 | 0.1716 | 0.1760 |
| ***Hmox1*** | 0.0264 | 0.0369 | 0.0323 | 0.0820 | 0.1191 | 0.1369 | 0.2862 | 0.2803 |
| ***Igkv1-110*** | 0.0603 | 0.1055 | 0.0558 | 0.0685 | 0.1949 | 0.1853 | 0.1508 | 0.1789 |
| ***Igkv5-43*** | 0.0483 | 0.0778 | 0.0357 | 0.0738 | 0.2058 | 0.2072 | 0.1773 | 0.1739 |
| ***LOC544905*** | 0.0671 | 0.0988 | 0.0619 | 0.0916 | 0.1761 | 0.1742 | 0.1618 | 0.1684 |
| ***Ifitm2*** | 0.0575 | 0.0653 | 0.0503 | 0.0670 | 0.1079 | 0.1546 | 0.2795 | 0.2180 |
| ***Ighv9-2*** | 0.0613 | 0.0955 | 0.0597 | 0.0923 | 0.1789 | 0.1837 | 0.1631 | 0.1656 |
| ***Igkv4-70*** | 0.0594 | 0.0959 | 0.0543 | 0.0894 | 0.2042 | 0.1927 | 0.1463 | 0.1577 |
| ***Igkv1-131*** | 0.0493 | 0.0846 | 0.0682 | 0.0910 | 0.1450 | 0.1606 | 0.2065 | 0.1948 |
| ***Ighv14-3*** | 0.0474 | 0.0827 | 0.0363 | 0.0722 | 0.2278 | 0.2008 | 0.1655 | 0.1673 |
| ***Ighv2-6-8*** | 0.0470 | 0.0857 | 0.0436 | 0.0724 | 0.1949 | 0.1839 | 0.1739 | 0.1988 |
| ***Igkv4-80*** | 0.0200 | 0.0672 | 0.0166 | 0.0436 | 0.3060 | 0.2899 | 0.1248 | 0.1318 |
| ***Igh-V3660*** | 0.0711 | 0.1051 | 0.0591 | 0.0874 | 0.1957 | 0.1793 | 0.1422 | 0.1601 |
| ***Igkv16-104*** | 0.0597 | 0.0945 | 0.0540 | 0.0847 | 0.1787 | 0.1789 | 0.1816 | 0.1679 |
| ***Ighv11-1*** | 0.0344 | 0.0726 | 0.0343 | 0.0713 | 0.2141 | 0.1951 | 0.1771 | 0.2010 |
| ***Igkv4-61*** | 0.0512 | 0.0949 | 0.0499 | 0.0804 | 0.2219 | 0.1913 | 0.1416 | 0.1688 |
| ***Iglv1*** | 0.0499 | 0.0886 | 0.0524 | 0.0864 | 0.1805 | 0.1837 | 0.1788 | 0.1796 |
| ***Igkv8-28*** | 0.0463 | 0.0804 | 0.0394 | 0.0792 | 0.2091 | 0.1890 | 0.1722 | 0.1844 |
| ***Ighv1-78*** | 0.0263 | 0.0623 | 0.0200 | 0.0531 | 0.2272 | 0.2142 | 0.1926 | 0.2044 |
| ***Igh-VS107*** | 0.0193 | 0.0502 | 0.0169 | 0.0534 | 0.2234 | 0.2137 | 0.1984 | 0.2248 |
| ***Igh-V11*** | 0.0301 | 0.0692 | 0.0282 | 0.0644 | 0.2091 | 0.2071 | 0.1899 | 0.2019 |
| ***Igkv4-74*** | 0.0626 | 0.1080 | 0.0547 | 0.0810 | 0.2105 | 0.1934 | 0.1320 | 0.1579 |
| ***Igh-VJ558*** | 0.0526 | 0.0929 | 0.0551 | 0.0769 | 0.1591 | 0.1631 | 0.1958 | 0.2044 |
| ***Vcam1*** | 0.0282 | 0.0423 | 0.0311 | 0.0757 | 0.1123 | 0.1298 | 0.3051 | 0.2756 |
| ***Ighv1-42*** | 0.0613 | 0.0958 | 0.0508 | 0.0899 | 0.2000 | 0.1819 | 0.1588 | 0.1614 |
| ***LOC102642252*** | 0.0481 | 0.0718 | 0.0315 | 0.0783 | 0.1943 | 0.2115 | 0.1827 | 0.1818 |
| ***Ighv10-3*** | 0.0436 | 0.0805 | 0.0358 | 0.0774 | 0.2096 | 0.1966 | 0.1628 | 0.1935 |
| ***Igkv2-109*** | 0.0611 | 0.0994 | 0.0582 | 0.0871 | 0.1726 | 0.1657 | 0.1777 | 0.1783 |
| ***Igkv4-69*** | 0.0573 | 0.1055 | 0.0582 | 0.0878 | 0.2043 | 0.1822 | 0.1453 | 0.1595 |
| ***Aif1*** | 0.0481 | 0.0593 | 0.0583 | 0.0999 | 0.1051 | 0.1297 | 0.2774 | 0.2221 |
| ***Adck1*** | 0.0331 | 0.0786 | 0.0297 | 0.0646 | 0.1953 | 0.1959 | 0.2060 | 0.1968 |
| ***Lgals1*** | 0.0720 | 0.1038 | 0.0614 | 0.0964 | 0.1683 | 0.1845 | 0.1735 | 0.1401 |
| ***Igkv3-4*** | 0.0658 | 0.1036 | 0.0599 | 0.0933 | 0.1618 | 0.1834 | 0.1715 | 0.1607 |
| ***Ighv14-2*** | 0.0432 | 0.0933 | 0.0601 | 0.0925 | 0.1610 | 0.1793 | 0.1762 | 0.1944 |
| ***Tgfbi*** | 0.0463 | 0.0535 | 0.0547 | 0.1069 | 0.1074 | 0.1063 | 0.2876 | 0.2373 |
| ***Ighv1-4*** | 0.0523 | 0.1010 | 0.0624 | 0.0715 | 0.1374 | 0.1940 | 0.1852 | 0.1963 |
| ***Ighv10-1*** | 0.0570 | 0.0991 | 0.0490 | 0.0903 | 0.1798 | 0.1831 | 0.1683 | 0.1734 |
| ***Igkv4-59*** | 0.0580 | 0.1219 | 0.0504 | 0.0718 | 0.2100 | 0.2048 | 0.1555 | 0.1275 |
| ***Igkv8-19*** | 0.0368 | 0.0826 | 0.0389 | 0.0838 | 0.1778 | 0.1893 | 0.1746 | 0.2163 |
| ***Igkv12-41*** | 0.0478 | 0.0840 | 0.0359 | 0.0832 | 0.2315 | 0.1741 | 0.1438 | 0.1998 |
| ***Ighv1-83*** | 0.0476 | 0.0954 | 0.0471 | 0.0876 | 0.2080 | 0.1684 | 0.1550 | 0.1909 |
| ***Igkv12-38*** | 0.0567 | 0.1096 | 0.0536 | 0.0887 | 0.1744 | 0.1955 | 0.1668 | 0.1546 |
| ***Igkv14-126*** | 0.0517 | 0.0915 | 0.0376 | 0.0835 | 0.1806 | 0.1818 | 0.1877 | 0.1856 |
| ***Snord69*** | 0.1974 | 0.2305 | 0.1460 | 0.1459 | 0.0631 | 0.0819 | 0.0641 | 0.0712 |
| ***Gm25128*** | 0.1857 | 0.1827 | 0.1873 | 0.1450 | 0.0778 | 0.0706 | 0.0754 | 0.0755 |
| ***Snord32a*** | 0.1776 | 0.1745 | 0.1829 | 0.1520 | 0.0678 | 0.0824 | 0.0839 | 0.0788 |
| ***Gm24613*** | 0.2132 | 0.1994 | 0.1822 | 0.1433 | 0.0619 | 0.0733 | 0.0517 | 0.0751 |
| ***Gm26293*** | 0.1420 | 0.1966 | 0.1585 | 0.2011 | 0.0628 | 0.0649 | 0.0809 | 0.0932 |
| ***Snord42b*** | 0.1417 | 0.2436 | 0.1460 | 0.2099 | 0.0463 | 0.0412 | 0.0838 | 0.0875 |
| ***1110038B12Rik*** | 0.1296 | 0.3000 | 0.1545 | 0.2283 | 0.0340 | 0.0294 | 0.0536 | 0.0705 |
| ***Gm25091*** | 0.2201 | 0.2045 | 0.1711 | 0.1287 | 0.0571 | 0.0846 | 0.0552 | 0.0788 |
| ***Gm24299*** | 0.2049 | 0.1921 | 0.1910 | 0.1242 | 0.0770 | 0.1040 | 0.0542 | 0.0526 |
| ***Gm25394*** | 0.1467 | 0.2304 | 0.1266 | 0.2994 | 0.0403 | 0.0418 | 0.0470 | 0.0677 |
| ***Gm26330*** | 0.1471 | 0.1866 | 0.1420 | 0.2829 | 0.0607 | 0.0541 | 0.0648 | 0.0619 |
| ***Snora74a*** | 0.1046 | 0.1836 | 0.1731 | 0.2648 | 0.0418 | 0.0544 | 0.0950 | 0.0828 |
| ***Scarna9*** | 0.1507 | 0.2522 | 0.1378 | 0.1705 | 0.0852 | 0.0864 | 0.0485 | 0.0686 |
| ***Gm23925*** | 0.1020 | 0.1584 | 0.1772 | 0.2775 | 0.0441 | 0.0610 | 0.0815 | 0.0983 |
| ***1110038B12Rik*** | 0.1185 | 0.1891 | 0.1723 | 0.2313 | 0.0772 | 0.0578 | 0.0592 | 0.0946 |
| ***Creld2*** | 0.0843 | 0.0832 | 0.0845 | 0.0824 | 0.1577 | 0.1656 | 0.1707 | 0.1717 |
| ***Cadm1*** | 0.0779 | 0.0751 | 0.0782 | 0.0784 | 0.1140 | 0.1201 | 0.2388 | 0.2175 |
| ***Igf1*** | 0.0724 | 0.0767 | 0.0857 | 0.0909 | 0.1286 | 0.1242 | 0.2087 | 0.2127 |
| ***Slc12a2*** | 0.0867 | 0.0947 | 0.0822 | 0.0886 | 0.1140 | 0.1109 | 0.2144 | 0.2085 |
| ***Postn*** | 0.0659 | 0.0747 | 0.0737 | 0.0787 | 0.0985 | 0.0951 | 0.2690 | 0.2444 |
| ***Mpzl1*** | 0.0629 | 0.0700 | 0.0593 | 0.0684 | 0.1145 | 0.1073 | 0.2496 | 0.2680 |
| ***Cd5l*** | 0.0725 | 0.0672 | 0.0765 | 0.0871 | 0.1092 | 0.1090 | 0.2314 | 0.2472 |
| ***Itgb5*** | 0.0656 | 0.0676 | 0.0726 | 0.0810 | 0.1117 | 0.1276 | 0.2353 | 0.2387 |
| ***Fcgr3*** | 0.0678 | 0.0738 | 0.0857 | 0.0938 | 0.1104 | 0.1121 | 0.2196 | 0.2368 |
| ***Fcgr4*** | 0.0711 | 0.0713 | 0.0720 | 0.0894 | 0.1236 | 0.1319 | 0.2288 | 0.2118 |
| ***Ckb*** | 0.0790 | 0.0849 | 0.0788 | 0.0874 | 0.1440 | 0.1385 | 0.2086 | 0.1788 |
| ***Gm11710*** | 0.0571 | 0.0636 | 0.0586 | 0.0791 | 0.1136 | 0.1150 | 0.2528 | 0.2602 |
| ***Prdm1*** | 0.1004 | 0.0890 | 0.0820 | 0.0689 | 0.1656 | 0.1574 | 0.1702 | 0.1665 |
| ***C6*** | 0.0744 | 0.0603 | 0.0915 | 0.0831 | 0.1054 | 0.1199 | 0.2227 | 0.2427 |
| ***Igkv1-99*** | 0.0930 | 0.0895 | 0.0937 | 0.0797 | 0.1272 | 0.1317 | 0.1774 | 0.2078 |
| ***Ptgs1*** | 0.0801 | 0.0779 | 0.0805 | 0.1002 | 0.1272 | 0.1332 | 0.2089 | 0.1919 |
| ***Pilrb1*** | 0.0518 | 0.0673 | 0.0619 | 0.0747 | 0.0920 | 0.1163 | 0.2527 | 0.2833 |
| ***Clec4a1*** | 0.0751 | 0.0909 | 0.0781 | 0.0940 | 0.1241 | 0.1210 | 0.2139 | 0.2028 |
| ***Mertk*** | 0.0577 | 0.0737 | 0.0748 | 0.0856 | 0.1059 | 0.1220 | 0.2562 | 0.2240 |
| ***Igsf6*** | 0.0559 | 0.0469 | 0.0598 | 0.0638 | 0.1077 | 0.0909 | 0.2307 | 0.3443 |
| ***Tgm1*** | 0.0778 | 0.0721 | 0.0915 | 0.1130 | 0.1113 | 0.1152 | 0.2035 | 0.2156 |
| ***Siglece*** | 0.0858 | 0.0760 | 0.0758 | 0.0973 | 0.1252 | 0.1314 | 0.2045 | 0.2041 |
| ***Cpq*** | 0.0693 | 0.0716 | 0.0846 | 0.0996 | 0.1373 | 0.1270 | 0.1864 | 0.2242 |
| ***P2ry13*** | 0.0646 | 0.0667 | 0.0547 | 0.0731 | 0.1292 | 0.1061 | 0.2885 | 0.2170 |
| ***Snora62*** | 0.0700 | 0.0859 | 0.0824 | 0.0764 | 0.0675 | 0.0581 | 0.2360 | 0.3236 |
| ***Fpr1*** | 0.0709 | 0.0660 | 0.0763 | 0.1013 | 0.1031 | 0.0998 | 0.2604 | 0.2221 |
| ***A530099J19Rik*** | 0.0923 | 0.0742 | 0.0897 | 0.0777 | 0.1167 | 0.1350 | 0.2171 | 0.1973 |
| ***Hfe*** | 0.0762 | 0.0858 | 0.0938 | 0.0978 | 0.1080 | 0.1281 | 0.1882 | 0.2222 |
| ***Sort1*** | 0.0705 | 0.0835 | 0.0870 | 0.0754 | 0.1255 | 0.0932 | 0.2401 | 0.2248 |
| ***Ccl6*** | 0.0966 | 0.0946 | 0.0831 | 0.1056 | 0.1204 | 0.1192 | 0.1979 | 0.1827 |
| ***Cd302*** | 0.0811 | 0.0865 | 0.0823 | 0.1024 | 0.1249 | 0.1219 | 0.2176 | 0.1835 |
| ***Ifi202b*** | 0.0952 | 0.0896 | 0.0875 | 0.1024 | 0.1096 | 0.1094 | 0.2268 | 0.1793 |
| ***Cmbl*** | 0.0733 | 0.0851 | 0.0764 | 0.0965 | 0.1189 | 0.0931 | 0.2404 | 0.2163 |
| ***Gm11710*** | 0.0718 | 0.0841 | 0.0715 | 0.1006 | 0.1111 | 0.1202 | 0.2117 | 0.2291 |
| ***Ighv1-5*** | 0.0878 | 0.0930 | 0.0673 | 0.0906 | 0.1461 | 0.1691 | 0.1781 | 0.1682 |
| ***Cd163*** | 0.0693 | 0.0881 | 0.0778 | 0.0919 | 0.1280 | 0.1087 | 0.2432 | 0.1931 |
| ***Tmem141*** | 0.0716 | 0.0927 | 0.0999 | 0.0746 | 0.1145 | 0.1216 | 0.2119 | 0.2132 |
| ***Hpgd*** | 0.0586 | 0.0716 | 0.0604 | 0.0933 | 0.0886 | 0.1124 | 0.2668 | 0.2483 |
| ***Fpr2*** | 0.0538 | 0.0697 | 0.0600 | 0.0886 | 0.1107 | 0.1273 | 0.2188 | 0.2711 |
| ***Mafb*** | 0.0777 | 0.0649 | 0.0820 | 0.1053 | 0.1426 | 0.1264 | 0.2087 | 0.1923 |
| ***Ighv1-59*** | 0.0700 | 0.1050 | 0.0607 | 0.0737 | 0.1505 | 0.1581 | 0.2049 | 0.1772 |
| ***Dnase1l3*** | 0.0815 | 0.0876 | 0.0754 | 0.0971 | 0.1441 | 0.1340 | 0.1676 | 0.2126 |
| ***Vstm4*** | 0.0799 | 0.0974 | 0.0826 | 0.0936 | 0.1202 | 0.1130 | 0.2376 | 0.1756 |
| ***Hebp1*** | 0.0591 | 0.0742 | 0.0672 | 0.1012 | 0.1192 | 0.1041 | 0.2412 | 0.2338 |
| ***Scarb1*** | 0.0848 | 0.1147 | 0.0857 | 0.1008 | 0.1128 | 0.1114 | 0.1970 | 0.1929 |
| ***AF251705*** | 0.0691 | 0.0743 | 0.0739 | 0.1037 | 0.1065 | 0.1353 | 0.2114 | 0.2258 |
| ***Tgm2*** | 0.0720 | 0.0700 | 0.0777 | 0.1162 | 0.1092 | 0.1128 | 0.2210 | 0.2211 |
| ***Rab3il1*** | 0.0760 | 0.0811 | 0.0803 | 0.1110 | 0.1051 | 0.1281 | 0.2100 | 0.2083 |
| ***Ighv8-5*** | 0.0441 | 0.0665 | 0.0431 | 0.0781 | 0.0950 | 0.0867 | 0.3393 | 0.2471 |
| ***Tspan4*** | 0.0879 | 0.0720 | 0.0875 | 0.1011 | 0.1256 | 0.1320 | 0.2230 | 0.1710 |
| ***Creg1*** | 0.0790 | 0.0854 | 0.0630 | 0.0985 | 0.1442 | 0.1260 | 0.2022 | 0.2018 |
| ***Epb4.1l3*** | 0.0941 | 0.0847 | 0.0879 | 0.1154 | 0.1083 | 0.1014 | 0.2234 | 0.1849 |
| ***Ubd*** | 0.0885 | 0.0985 | 0.0824 | 0.1111 | 0.1198 | 0.1057 | 0.2097 | 0.1842 |
| ***Clec4b1*** | 0.0602 | 0.0564 | 0.0559 | 0.1025 | 0.1139 | 0.1054 | 0.2496 | 0.2561 |
| ***Pla2g2d*** | 0.0912 | 0.1093 | 0.0725 | 0.0984 | 0.1396 | 0.1479 | 0.1575 | 0.1837 |
| ***Tnfrsf21*** | 0.0700 | 0.1002 | 0.0762 | 0.1051 | 0.1211 | 0.1209 | 0.2221 | 0.1845 |
| ***Igkv4-78*** | 0.0770 | 0.1051 | 0.0653 | 0.0902 | 0.1748 | 0.1766 | 0.1573 | 0.1537 |
| ***Sirpa*** | 0.0639 | 0.0928 | 0.0817 | 0.1073 | 0.1116 | 0.1355 | 0.2000 | 0.2074 |
| ***App*** | 0.0710 | 0.0856 | 0.0757 | 0.1147 | 0.1166 | 0.0963 | 0.1890 | 0.2510 |
| ***Sdc3*** | 0.0627 | 0.0617 | 0.1254 | 0.0723 | 0.1307 | 0.1076 | 0.2223 | 0.2172 |
| ***Igkv14-130*** | 0.0647 | 0.1008 | 0.0517 | 0.0928 | 0.1463 | 0.1527 | 0.2019 | 0.1892 |
| ***Ighv1-31*** | 0.0632 | 0.1139 | 0.0469 | 0.0724 | 0.1199 | 0.1392 | 0.1569 | 0.2874 |
| ***Sh2d1b1*** | 0.0795 | 0.1281 | 0.0868 | 0.0985 | 0.1191 | 0.1131 | 0.1708 | 0.2042 |
| ***Igkv9-124*** | 0.0576 | 0.0953 | 0.0463 | 0.0889 | 0.1747 | 0.1571 | 0.1806 | 0.1996 |
| ***n-R5s89*** | 0.1044 | 0.1245 | 0.0762 | 0.1204 | 0.0857 | 0.0869 | 0.2311 | 0.1709 |
| ***Il18*** | 0.0748 | 0.1016 | 0.0556 | 0.1107 | 0.1293 | 0.1236 | 0.2031 | 0.2012 |
| ***Igh-VJ558*** | 0.0743 | 0.1121 | 0.0499 | 0.0889 | 0.1787 | 0.1692 | 0.1571 | 0.1699 |
| ***Acp2*** | 0.0826 | 0.0753 | 0.0652 | 0.1195 | 0.1071 | 0.1258 | 0.2438 | 0.1807 |
| ***Gm25183*** | 0.0805 | 0.0856 | 0.1042 | 0.0524 | 0.0788 | 0.1430 | 0.2311 | 0.2244 |
| ***Ighv9-1*** | 0.0738 | 0.1057 | 0.0670 | 0.1082 | 0.1348 | 0.1524 | 0.1764 | 0.1818 |
| ***Igk-V28*** | 0.0706 | 0.1073 | 0.0589 | 0.0963 | 0.1664 | 0.1752 | 0.1680 | 0.1571 |
| ***Ighv1-42*** | 0.0613 | 0.0958 | 0.0508 | 0.0899 | 0.2000 | 0.1819 | 0.1588 | 0.1614 |
| ***Igkv1-132*** | 0.0303 | 0.0706 | 0.0207 | 0.0719 | 0.1912 | 0.1565 | 0.2297 | 0.2290 |
| ***Gm25246*** | 0.1046 | 0.1012 | 0.0842 | 0.0739 | 0.1308 | 0.1260 | 0.2536 | 0.1259 |
| ***Ctsb*** | 0.0678 | 0.1012 | 0.0627 | 0.1134 | 0.1325 | 0.1388 | 0.1876 | 0.1960 |
| ***Ighv1-61*** | 0.0709 | 0.0998 | 0.0410 | 0.0870 | 0.2114 | 0.1772 | 0.1633 | 0.1494 |
| ***Igkv1-122*** | 0.0766 | 0.1141 | 0.0623 | 0.1003 | 0.1650 | 0.1648 | 0.1564 | 0.1605 |
| ***Gbp8*** | 0.0585 | 0.0780 | 0.1024 | 0.0844 | 0.1488 | 0.0858 | 0.2652 | 0.1768 |
| ***Cfp*** | 0.0525 | 0.0939 | 0.0766 | 0.1159 | 0.1214 | 0.1232 | 0.2179 | 0.1985 |
| ***Igkv8-18*** | 0.0559 | 0.0983 | 0.0561 | 0.0996 | 0.1854 | 0.1425 | 0.1768 | 0.1854 |
| ***Tyrobp*** | 0.0634 | 0.1032 | 0.0533 | 0.1145 | 0.1281 | 0.1361 | 0.2064 | 0.1950 |
| ***Lst1*** | 0.0477 | 0.0932 | 0.0578 | 0.1212 | 0.0871 | 0.1096 | 0.2632 | 0.2203 |
| ***Spic*** | 0.0680 | 0.1185 | 0.0611 | 0.1152 | 0.1176 | 0.1148 | 0.2222 | 0.1827 |
| ***Igkv4-53*** | 0.0569 | 0.1348 | 0.0515 | 0.0942 | 0.1328 | 0.1369 | 0.1973 | 0.1956 |
| ***Gm25633*** | 0.1343 | 0.1217 | 0.1786 | 0.1679 | 0.1203 | 0.1215 | 0.0816 | 0.0742 |
| ***Gm10012*** | 0.1756 | 0.1586 | 0.1688 | 0.1613 | 0.1131 | 0.1017 | 0.0547 | 0.0662 |
| ***LOC102632389*** | 0.1451 | 0.1383 | 0.1819 | 0.1685 | 0.1062 | 0.0888 | 0.0815 | 0.0896 |
| ***LOC102632389*** | 0.1451 | 0.1383 | 0.1819 | 0.1685 | 0.1062 | 0.0888 | 0.0815 | 0.0896 |
| ***Snord57*** | 0.1869 | 0.1803 | 0.1582 | 0.1430 | 0.1016 | 0.0887 | 0.0645 | 0.0768 |
| ***Snora81*** | 0.1601 | 0.1131 | 0.2049 | 0.2277 | 0.1096 | 0.0810 | 0.0536 | 0.0500 |
| ***Snord100*** | 0.1885 | 0.1629 | 0.1826 | 0.1461 | 0.0862 | 0.0916 | 0.0741 | 0.0679 |
| ***Gm26397*** | 0.1299 | 0.1770 | 0.1636 | 0.1717 | 0.0990 | 0.0985 | 0.0847 | 0.0757 |
| ***Snord47*** | 0.1701 | 0.1179 | 0.2184 | 0.1386 | 0.1246 | 0.1120 | 0.0650 | 0.0535 |
| ***Atg16l1*** | 0.1532 | 0.1351 | 0.1692 | 0.2089 | 0.0683 | 0.1003 | 0.0887 | 0.0763 |
| ***Gm23297*** | 0.1538 | 0.1902 | 0.1871 | 0.1552 | 0.0798 | 0.0919 | 0.0857 | 0.0562 |
| ***Gm26225*** | 0.1316 | 0.0980 | 0.4011 | 0.1410 | 0.0720 | 0.0773 | 0.0457 | 0.0334 |
| ***Gm23503*** | 0.0796 | 0.1661 | 0.1776 | 0.2737 | 0.0923 | 0.0817 | 0.0523 | 0.0767 |
| ***Gm24411*** | 0.1692 | 0.1100 | 0.1498 | 0.1868 | 0.1021 | 0.1185 | 0.0727 | 0.0909 |
| ***Gm26184*** | 0.0897 | 0.1294 | 0.1846 | 0.2596 | 0.0666 | 0.0779 | 0.1144 | 0.0779 |
| ***Gm24336*** | 0.0974 | 0.1394 | 0.1885 | 0.1805 | 0.0895 | 0.1203 | 0.0756 | 0.1087 |
| ***Gm25559*** | 0.1854 | 0.0853 | 0.2457 | 0.1362 | 0.0917 | 0.1547 | 0.0585 | 0.0426 |
| ***Gm22574*** | 0.2089 | 0.0934 | 0.1591 | 0.1268 | 0.1798 | 0.1298 | 0.0550 | 0.0472 |
| ***mt-Tf*** | 0.1113 | 0.1653 | 0.1396 | 0.2104 | 0.1004 | 0.1114 | 0.0665 | 0.0951 |
| ***Snord87*** | 0.1699 | 0.1356 | 0.2590 | 0.1060 | 0.1248 | 0.1067 | 0.0656 | 0.0325 |
| ***Gm25617*** | 0.0635 | 0.1317 | 0.1856 | 0.3213 | 0.0535 | 0.0548 | 0.1005 | 0.0893 |
